# Prospective Randomized Trial of Enoxaparin, Pentoxifylline and Ursodeoxycholic Acid for Prevention of Radiation-Induced Liver Toxicity

**DOI:** 10.1371/journal.pone.0112731

**Published:** 2014-11-13

**Authors:** Max Seidensticker, Ricarda Seidensticker, Robert Damm, Konrad Mohnike, Maciej Pech, Bruno Sangro, Peter Hass, Peter Wust, Siegfried Kropf, Günther Gademann, Jens Ricke

**Affiliations:** 1 Universitätsklinik Magdeburg, Klinik für Radiologie und Nuklearmedizin, Magdeburg, Germany; 2 International School of Image-Guided Interventions, Deutsche Akademie für Mikrotherapie, Magdeburg, Germany; 3 Clinica Universidad de Navarra, Liver Unit, Department of Internal Medicine, Pamplona, Spain; 4 Universitätsklinik Magdeburg, Klinik für Strahlentherapie, Magdeburg, Germany; 5 Charité Universitätsmedizin Berlin, Klinik für Radioonkologie und Strahlentherapie, Berlin, Germany; 6 Universitätsklinik Magdeburg, Institut für Biometrie und Medizinische Informatik, Magdeburg, Germany; 7 Medical University of Gdansk, 2nd Department of Radiology, Gdansk, Poland; The Chinese University of Hong Kong, Hong Kong

## Abstract

**Background/Aim:**

Targeted radiotherapy of liver malignancies has found to be effective in selected patients. A key limiting factor of these therapies is the relatively low tolerance of the liver parenchyma to radiation. We sought to assess the preventive effects of a combined regimen of pentoxifylline (PTX), ursodeoxycholic acid (UDCA) and low-dose low molecular weight heparin (LMWH) on focal radiation-induced liver injury (fRILI).

**Methods and Materials:**

Patients with liver metastases from colorectal carcinoma who were scheduled for local ablation by radiotherapy (image-guided high-dose-rate interstitial brachytherapy) were prospectively randomized to receive PTX, UDCA and LMWH for 8 weeks (treatment) or no medication (control). Focal RILI at follow-up was assessed using functional hepatobiliary magnetic resonance imaging (MRI). A minimal threshold dose, i.e. the dose to which the outer rim of the fRILI was formerly exposed to, was quantified by merging MRI and dosimetry data.

**Results:**

Results from an intended interim-analysis made a premature termination necessary. Twenty-two patients were included in the per-protocol analysis. Minimal mean hepatic threshold dose 6 weeks after radiotherapy (primary endpoint) was significantly higher in the study treatment-group compared with the control (19.1 Gy versus 14.6 Gy, p = 0.011). Qualitative evidence of fRILI by MRI at 6 weeks was observed in 45.5% of patients in the treatment versus 90.9% of the control group. No significant differences between the groups were observed at the 12-week follow-up.

**Conclusions:**

The post-therapeutic application of PTX, UDCA and low-dose LMWH significantly reduced the extent and incidence fRILI at 6 weeks after radiotherapy. The development of subsequent fRILI at 12 weeks (4 weeks after cessation of PTX, UDCA and LMWH during weeks 1–8) in the treatment group was comparable to the control group thus supporting the observation that the agents mitigated fRILI.

**Trial Registration:**

EU clinical trials register 2008-002985-70 ClinicalTrials.gov NCT01149304

## Introduction

Highly targeted radiotherapy of liver malignancies has found to be effective in selected patients. Stereotactic radiotherapy, radioembolization using yttrium-90 (^90^Y) microspheres as well as image-guided brachytherapy (BT) have been described in the literature with promising results [1], [2], [3]. A key limiting factor of these therapies is the relatively low tolerance of the liver parenchyma to radiation leading to either subclinical focal or generalized injury of the liver parenchyma. When the intensity or the extent of radiation-induced liver injury (RILI) exceeds the functional reserve, clinical complications appear in the form of radiation (radioembolization) induced liver disease (RILD or REILD) [4], [5], [6], [7]. Prior exposure or concomitant chemotherapy is thought to increase the risk of RILD (or REILD), and as a consequence is a relatively common complication, for example, after conditioning therapy prior to bone marrow transplantation (BMT) [5], [8], [9], [10]. Liver damage whether associated with whole body irradiation or liver-directed radiotherapy have the same pathology, i.e. veno-occlusive disease (VOD) [5], [11], [12], [13].

Medication designed to reduce RILI could improve the safety as well as enable more aggressive radiotherapy. Clinical studies have shown with varying strength of evidence that VOD/RILD after BMT can be ameliorated by pentoxifylline (PTX), ursodeoxycholic acid (UDCA) and low molecular weight heparin (LMWH) [14], [15], [16], [17], [18], [19], [20], [21], [22] (see Table 1). However, the equivocal nature of the results from most studies probably reflect the heterogeneous study populations (including patients who have received prior chemotherapy or had underlying liver disease) [23]. Thus, a more standardized clinical model is needed to evaluate the protective effects of prophylactic regimens against VOD/RILD.

**Table 1:** Summary of published studies on drug treatments for the prevention of VOD/RILD.

Reference	Studydesign	N	Treatment regimen	Incidenceof VOD	p-value*	Bilirubin(µmol/L)	p-value*
Attal et al.1993 [14]	ProspectiveRCT	70	**Pentoxifylline** 1,600 mg/dday −8 to day+100 post-BMT	4%	NS	26.4(mean max)	NS
		70	Control	3%		24.4(mean max)	
Clift et al.1993 [22]	ProspectiveRCT	44	**Pentoxifylline** 2,400 mg/dday −3 to day+70 post-allogeneic BMT	-		26.6(mean max)	0.62
		44	Control	-		23.47(mean max)	
Bianco et al.1991 [16]	Phase 1–2	30	**Pentoxifylline** 1,200, 1,600,and 2,000 mg/d; day−10 to day+100 post-BMT	10%	0.001	-	-
		20	Control (retrospective)	65%		-	
Attal et al1992 [15]	ProspectiveRCT	81	**Unfractionated heparin**100 U/kg/d cont. infusion;day −8 to day+30 post-BMT	2.5%	0.01	7.4%exceeding 34	<0.05
		80	Control	14%		18.7%exceeding 34	
Forrest et al.2003 (18)	Prospectivesingle-arm	40	**LMWH**: dalteparin 2500anti-Xa i.u; day −1 to day+30 post-BMT or hospitaldischarge	22.5%, 2.5% severe			
Or et al.1996 [20]	ProspectiveRCT, pilot	61	**LMWH**: enoxaparin40 mg/day; day+1to day+40 post-BMTor hospital discharge		0.01	(duration ofelevatedlevels)	0.01
		33	Control				
Essel et al.1998 [17]	ProspectiveRCT	34	**UDCA** 600–1200 mg/d;day at least −1 to day+80 post-BMT	15%	0.03	102.6(mean max)	0.13
		32	Control	40%		188.1(mean max)	
Ohashi et al.2000 [19]	ProspectiveRCT	67	**UDCA** 600 mg/d;day −21 to day+80post-BMT	3%	0.004	Not reportedin detail	NS
		65	Control	18.5%		Not reportedin detail	
Park et al.2002 [28]	ProspectiveRCT	82	**UDCA** 600 mg/d **+** **unfractionated heparin**5–50 U/kg/d adjusted aPTTof 50 s; day +1 to day +30post-BMT or hospitaldischarge (but aminimum of 15d)	16%	0.348	148.8(mean max)	0.725
		83	**Unfractionated heparin**5–50 U/kg/d adjustedaPTT of 50 s; day +1 today +30 post-BMT orhospital discharge(but a minimum of 15d)	19%		173.6(mean max)	

*Group comparison; LMWH: Low molecular weight heparin; BMT: Bone marrow transplantation; Max: Maximum; NS: Not significant; VOD: Veno-occlusive disease; RCT: Randomized controlled trial; UDCA: ursodeoxycholic acid (ursodiol); aPTT: activated Partial Thromboplastin Time.

**Figure 1:**
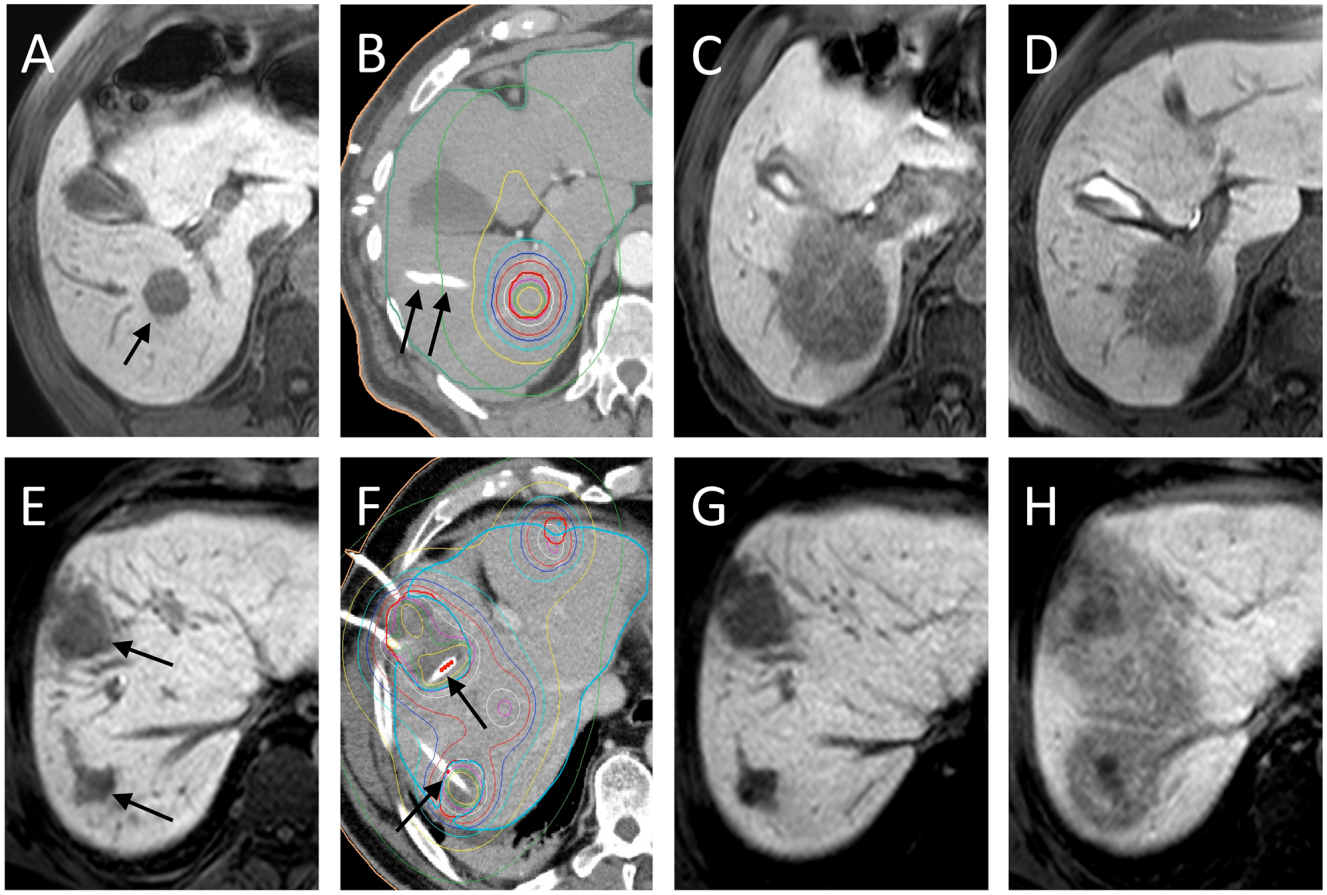
T1w-axial THRIVE 20 min after application of Gd-EOB-DTPA (A, C–E and G, H) and BT planning CT with dosimetry (B and F). A–D, control group. A: pre-treatment MRI displaying a metastasis scheduled for BT treatment (black arrow). B: Planning-CT after introduction of the brachytherapy catheters (black arrows). Clinical target volume (CTV) represented by bold red circle and dosimetry by coloured lines (red: 20 Gy-, blue: 12 Gy-isodose). C: MRI at 6 weeks showing substantial reduction in Gd-EOB-DTPA uptake by liver parenchyma adjacent to treated metastases (i.e. focal radiation-induced liver injury, fRILI). Note: The area of fRILI matches the geometry of the dosimetry (B). Determined threshold dose: 9.75 Gy. D: MRI at 3 months showing shrinkage of the fRILI. Determined threshold dose: 11.9 Gy. E–H, treatment group. E: pre-treatment MRI displaying two metastases (black arrow); two more treated lesions are not displayed in the plane. F: Planning-CT (annotations: see B). G: MRI at 6 weeks showing no fRILI. H: MRI at 3 months after radiotherapy (and 1 month after finishing study treatment) showing a substantial region of fRILI. Determined threshold dose: 15.8 Gy.

Image-guided, single-fractioned, high-dose-rate BT of liver malignancies is associated with a well-characterized focal RILI (fRILI), which can be visualized and quantified using functional hepatobiliary magnetic resonance imaging (MRI) (see Figure 1) [6], [7]. Importantly, the histopathological evidence of fRILI (i.e. sinusoidal congestion with hepatocyte atrophy and increased reticulin deposits) correlates well with the absence of the hepatocyte uptake of hepatolbiliary MRI contrast media [24]. We have previously found that development of areas of fRILI were maximal at 6–8 weeks post-BT which correlates to the peak incidence of RILD/REILD after conditioning therapy/radioembolization througout the first 2 months post-intervention [5], [6], [7], [25]. We conducted a prospective study to quantify fRILI in patients who were randomized to BT with and without prophylactic PTX, UDCA and low-dose LMWH. To minimize the confounding effects of prior chemotherapy on radiation tolerability, only patients with liver metastases from colorectal cancer (mCRC) were included because these patients tend to have a more consistent pattern of prior exposition to chemotherapy. The cumulative effect of three drugs over a period of 8 weeks [26], [27], [28] was assessed and patients followed-up at 6 and 12 weeks.

## Materials and Methods

The protocol for this trial and supporting CONSORT checklist are available as supporting information; see Checklist S1 and Protocol S1.

### Study design

This was a prospective, randomised phase II, parallel-group, open-label study conducted at a single centre. The study was approved by the competent authorities (Federal Institute for Drugs and Medical Devices (in german: Bundesinstitut für Arzneimittel und Medizinprodukte - BfArM)) and the local ethics committee (Ethikkommission der Otto-von-Guericke-Universität der Medizinischen Fakultät). Trial registration: Eudra-CT: 2008-002985-70; ClinicalTrials.gov-identifier NCT01149304. Written informed consent was obtained from all patients prior to study entry. Group allocation approach was unrestricted randomization.

### Patient characteristics

**Figure 2:**
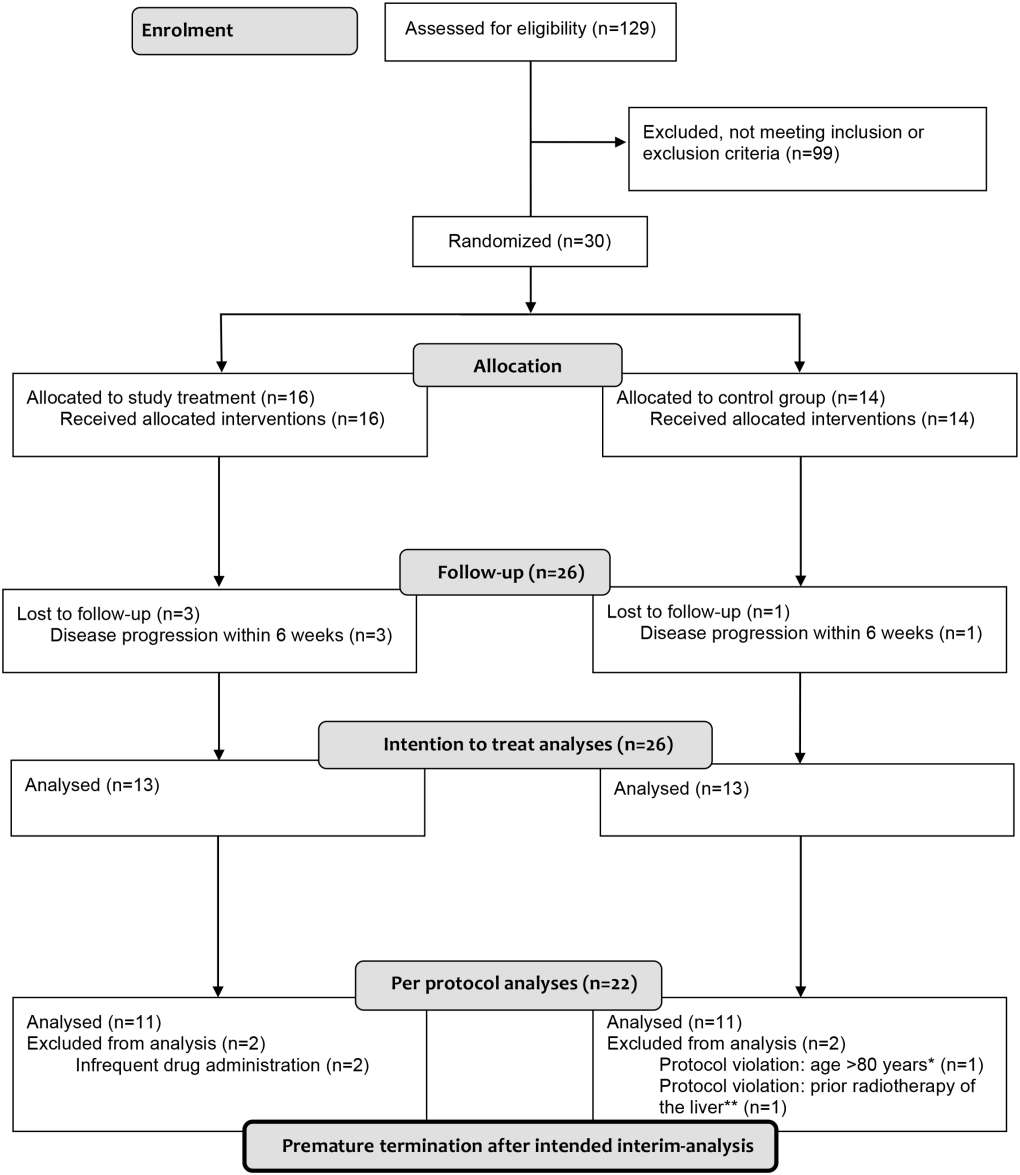
CONSORT-diagram. *Exclusion criterion age was initially disregarded by error in this patient (aged 82). **Exclusion criterion prior radiotherapy was initially disregarded by error in this patient (prior radiotherapy was performed 2 years earlier with location in the contralateral liver lobe).

Consecutive patients (18–80 years) with liver metastases from mCRC, who were scheduled for local ablation with computed-tomography (CT)/MRI-guided BT between 2009 and 2012, were screened (Figure 2). (BT is the local standard ablative treatment in patients ineligible for surgical or all other appropriate intervention).

Women who were pregnant, lactating or of childbearing potential were excluded as were patients with liver cirrhosis, hepatitis B or C, severe coronary artery disease, autoimmune diseases, acute bacterial endocarditis, active major bleedings or high-risk of uncontrolled hemorrhage; severe or moderate renal impairment (GFR <60 mL/min), or known contraindication or hypersensitivity to any of the study treatments or procedures.

### Treatment and follow-up

Patients received a single-fraction, CT- or MRI-guided BT of CRC liver metastases (see details below). In those randomized to prophylaxis, the following treatment was initiated during the evening of the day of BT: sc injection of 40 mg q.d. enoxaparin (Clexane, Sanofi Aventis, Paris, France) [20], oral 400 mg t.i.d. PTX (Trental, Sanofi Aventis) [16] and oral 250 mg t.i.d. UDCA (Ursofalk, Falk Pharma, Freiburg, Germany) [17], [19]. Patients were discharged usually on the third day post-BT and continued to take study medication at home for 8 weeks. All patients were followed-up on day 3, week 6 and 12 with an optional follow-up at week 24. Within 24 hours of the procedure and at each subsequent visit, blood samples were taken for liver-specific and inflammatory/hemostatic laboratory parameters, and patients were assessed for ECOG-performance status and health-related quality-of-life (using the EQ5D-questionnaire). All adverse reactions related to the study medication or BT were recorded.

Compliance to the prophylactic regimen was evaluated during a dialogue at each visit and the evaluation of anti-Xa-activity at 6 weeks. Insufficient compliance was determined by: either anti-Xa-activity <0.1 IU/mL measured up to 4 hours after last enoxaparin injection, or two dose interruptions of the prophylactic regimen for more than 1 day/week. Non-compliant patients were withdrawn from the per-protocol analysis and study-specific medication stopped.

### Image-guided interstitial brachytherapy

The technique of image-guided BT has been described previously [2]. Briefly, the placement of the introducer sheaths (6F Radiofocus, Terumo, Tokyo, Japan) with the BT applicators (Lumencath, Nucletron/Elekta, Veenendaal, The Netherlands) was performed using CT or MRI fluoroscopy. For treatment planning purposes, a spiral CT or T1-weighted MRI of the liver (reconstructed slice thickness: 3 mm) enhanced by intravenous application of iodine contrast media (CT) or Gd-EOB-DTPA (MRI) was acquired.

The high-dose-rate afterloading system (Microselectron, Nucletron/Elekta, Veenendaal, The Netherlands) employed an iridium-192 source with a nominal activity of 10Ci (i.e. 370GBq); decay correction was performed daily. Relative coordinates (x, y, z) of the catheters were determined in the CT/MRI-data set and transferred to the treatment planning system (Oncentra, Nucletron/Elekta). Using these coordinates, the clinical target volume and the predefined minimum dose (20 Gy, delivered as a single fraction [2]), the software calculated a dosimetry and the duration of the iridium-192 source inside the BT catheters. A planning CT with dosimetry is displayed in Figure 1B and F.

### Magnetic resonance imaging

MRI (Achieva 1.5T, Philips, Best, The Netherlands) using the hepatobiliary contrast medium Gd-EOB-DTPA (Primovist, Bayer Healthcare, Leverkusen, Germany) was performed 1 day before and 6 and 12 weeks post-BT. MR-sequence of events was as follows: axial 3D T1-weighted (T1-w) gradient echo THRIVE (T1-High-Resolution-Isotropic-Volume-Excitation) (Time-to-Echo/Time-to-Repetition 4/10 ms, flip-angle 10°) with fat-suppression pre-contrast, at 20 s, 60 s and 120 s and 20 minutes after iv 0.1 mL/kg bodyweight Gd-EOB-DTPA. The slice thickness was 3 mm. For the study-specific MRI volumetry, dynamic THRIVE at 60 s (for the exclusion of tumor progression/local recurrence) and hepatobiliary phase THRIVE 20 min after application of Gd-EOB-DTPA (for the determination of area of fRILI) were mandatory.

Identification of the radiation isodose (minimal hepatic threshold dose) that demarcated the border between the fRILI and functioning liver tissue (as defined by non-uptake and uptake of Gd-EOB-DTPA enhanced MRI, respectively) was performed as follows in a blinded matter.

The hepatobiliary phase THRIVE was transferred to the BT-planning software. Image registration of the hepatobiliary phase THRIVE to the contrast-enhanced planning CT/MRI (including the dosimetry) was performed by an isoscalar local semi-automated point-based 3D-3D image registration using predefined match points (3 or 4 corresponding landmarks restricted to liver structures). Registration was only accepted if the target area merged perfectly by visual assessment. As a result of this procedure, the software simultaneously displayed the treatment dosimetry and anatomical structures/fRILI of the hepatobiliary phase THRIVE. The volume of the liver parenchyma with radiation-induced impaired uptake of Gd-EOB-DTPA (i.e. fRILI) was determined. The isodose of the dosimetry encircling this volume was determined at five different axial levels and the mean of these values recorded. This dose resembles the dose which was formerly applied at the now demarcated rim of the fRILI, corresponding to the assumed minimal hepatic tolerance dose. To ensure a negligible registration error, the volume of fRILI was inserted into the dose-volume-histogram of the dosimetry. The corresponding isodose was stored. Results of the two methods showed a high correlation of 0.899 and 0.562 (p<0.001 and p = 0.006) for 6 and 12 weeks, respectively. To minimize methodological errors, the mean isodose value of the two methods was taken. In case of more than one treated lesion, the mean of the determined isodoses was used. If no detectable fRILI was seen in follow-up, the minimal mean hepatic threshold dose was defined as the dose which was previously administered at the tumor margin (since an effect on the liver parenchyma above this dose level cannot be excluded). Figure 1 illustrates the development and appearance of the fRILI in hepatobiliary phase THRIVE.

### Endpoints and statistical analyses

The aim of the study was to assess if a combination regimen of PTX, UDCA and low-dose LMWH for 8 weeks provided a preventive effect regarding irradiation damage to liver parenchyma (as resembled by the minimal mean threshold dose of the fRILI volume) at 6 weeks (primary endpoint) and at 12 weeks (secondary endpoint) after BT.

As additional descriptor, detectable fRILI in Gd-EOB-DTPA MRI (yes/no) was recorded at each follow-up. Further secondary objectives included the safety of the study treatment after BT including changes in bilirubin and albumin which were graded according to Common Terminology Criteria for Adverse Events version 3 (CTCAE3.0).

The relation between hepatocyte dysfunction and changes in the following liver-specific and inflammatory/hemostatic laboratory values were analysed: fibrinogen, factor-VIII-activity, interleukin-6, protein-C-activity, protein-S-activity, von-Willebrand-factor-activity and antithrombin-III-activity [29].

Determination of sample size was based on the expected minimum between-group difference of 2.1 Gy (SD 2.3 Gy) for minimal mean hepatic threshold dose at 6 weeks after BT (from 9.9 Gy to 12 Gy) [7]. A sequential test with 2 stages according to the Pocock-design was used which yielded a total of 22 observations per group with a scheduled interim analysis after 11 observations per group when a = 0.025 and power 1-b = 0.8. Interim-analysis showed a significant difference between the groups regarding the primary variable with a one-sided p-value of 0.011. A one-sided p of <0.0148 was necessary to terminate the study prematurely.

Statistical analysis was performed using SPSS (SPSS21, IBM, Chicago, Il, USA). Descriptive analysis of patient characteristics and laboratory findings was performed. The primary analysis was evaluated in the per protocol cohort and repeated in the intention-to-treat population as sensitivity analysis. Between-group differences in minimal mean hepatic threshold after BT at 6 and 12 weeks were compared using a two-sample *t*-tests, and evidence of detectable fRILI were compared using the Fisher’s-exact-test. Possible confounding factors were evaluated using the Mann-Whitney-*U*-test for metric variables and the Fisher’s-exact-test for categorical variables, and then between-group differences for the primary endpoint were evaluated with inclusion of the covariables (ANOVA and ANCOVA). The relationship between the minimal mean hepatic threshold dose and laboratory values was tested by Pearson’s correlation and ANCOVA. Group comparison regarding ECOG and EQ5D was made by Mann-Whitney-*U*-test. Median overall survival was estimated by Kaplan-Meier (group comparison by log-rank test). A p-value of <0.05 was statistically significant.

## Results

Of 129 patients screened with liver metastases from colorectal cancer scheduled for BT, 30 patients were included in the study and 22 patients (11 per group) in the primary analyses of the per-protocol group (see CONSORT diagram, Figure 2). Demographic characteristics of randomized patients at screening are summarized in Table 2 and the baseline liver function and other laboratory parameters are presented in Table 3. Group comparison revealed a similar distribution of possible confounders. A tendency towards a larger volume of significantly radiation exposed liver parenchyma (>10 Gy) in the study treatment group (Table 2) may have potentially lowered the hepatic tolerance dose in this group instead of increase it [25].

**Table 2:** Patient characteristics (per protocol analysis).

Variable	Treatmentgroup (n = 11)	Control(n = 11)	p-value(betweengroup)*
Sex (m/f)	9/2	8/3	1.000
Age (years)	71.09±5.47	65.09±12.55	0.408
Weight (kg)	84.64±11.68	83.91±12.89	0.592
Height (cm)	174.09±6.79	172.64±6.90	0.834
ECOG at baseline (0/1/2)	6/4/1	4/5/2	0.370
EQ5D visual analogue score	72.36±14.56	76.36±13.02	0.446
History of liver surgery	45.5%	45.5%	1.000
Steatosis hepatis	36.4%	18.2%	0.635
Diabetes mellitus	18.2%	27.3%	1,000
Chemotherapy pretreatment			
Applied lines	1.00±0.63	1.00±0.45	1.000
no chemotherapy	18.2%	9.1%	NA
1 line	63.6%	81.8%	0.672
2 lines	18.2%	9.1%	NA
Prior chemotherapy			
Oxaliplatin	63.6%	63.6%	1.000
Irinotecan	36.4%	36.4%	1.000
Biologicals	54.5%	54.5%	1.000
Number of treated metastases	1.91±1.04	1.45±0.52	0.382
Maximum diameter of metastases (mm)	37.18±12.91	29.45±11.79	0.146
Clinical target volume (cm^3^)	42.82±29.26	31.36±37.14	0.156
Number of used brachytherapy catheters	3.18±1.78	2.27±1.74	0.079
Liver volume (cm^3^)	1296.1±226.6	1451.3±278.6	0.401
Interval between BT and 6 weeks FU (days)	43.91±4.76	45.09±4.68	0.757
Interval between BT and 3 months FU (days)	87.34±4.52	89.55±6.15	0.505
Liver volume with a dose exposure >10 Gy (%)	22.55±14.45	11.95±10.43	0.056
Chemotherapy during follow-up	18.2%	9.1%	1.000

Continuous data: mean ± standard deviation, frequencies: counts or percent.

*Group comparison, continuous data compared by Mann-Whitney U test, frequency data compared by Pearson’s chi square test.

**Table 3:** Laboratory parameters at baseline and follow-up (per protocol analysis).

Variable(normal range)		Treatmentgroup (n = 11)	Control(n = 11)	p-value(betweengroup)*	p-value(baseline *vs*.follow-up)**
Bilirubin	baseline	8.27±2.92	8.39±5.61	0.594	
(<21.0 µmol/L)	6 weeks	9.58±9.94	9.56±7.18	0.641	0.182 (0.350)
	12 weeks	8.71±4.27	8.75±5.95	0.735	0.594 (0.505)
Albumin	baseline	44.21±3.46	44.05±2.45	0.833	
(35.0–52.0 g/L)	6 weeks	42.49±5.16	42.67±3.17	0.743	0.197 (0.060)
	12 weeks	42.84±4.94	43.66±2.31	0.743	0.212 (0.332)
Cholinesterase	baseline	149.26±47.97	144.73±21.73	0.718	
(88–215 µmol/s.L)	6 weeks	136.27±51.65	143.82±29.10	0.433	**0.023** (0.929)
	12 weeks	132.94±49.22	153.36±30.96	0.088	**0.010** (0.423)
Aspartate transaminase	baseline	0.56±0.18	0.46±0.17	0.211	
(0.17–0.83 µmol/s.L)	6 weeks	0.59±0.17	0.55±0.23	0.533	0.373 (**0.016**)
	12 weeks	0.63±0.47	0.54±0.17	0.974	0.563 (0.056)
Alanine transaminase	baseline	0.44±0.20	0.51±0.36	1,000	
(0.17–0.83 µmol/s.L)	6 weeks	0.50±0.18	0.62±0.45	0.742	0.443 (0.109)
	12 weeks	0.53±0.43	0.52±0.27	0.718	0.508 (0.722)
Gamma glutamyltransferase	baseline	1.61±2.62	1.49±1.21	0.189	
(0.17–1.19 µmol/s.L)	6 weeks	0.82±0.83	2.21±1.71	**0.011**	0.100 (0.050)
	12 weeks	1.25±1.17	1.97±1.49	0.139	0.722 (0.306)
Glutamate dehydrogenase	baseline	104.36±91.47	108.82±94.84	0.844	
(<120 nmol/s.L)	6 weeks	67.55±31.43	123.27±105.88	0.490	0.328 (0.308)
	12 weeks	128.11±108.79	126.09±95.19	0.849	0.674 (0.374)
International normalized	baseline	93.9±3.03	95.55±2.98	0.053	
ratio (0.85–1.27)	6 weeks	94.11±2.71	94.8±2.44	0.399	0.438 (0.502)
	12 weeks	94.63±2.50	95.33±3.61	0.732	0.334 (0.498
Interleukin 6	baseline	4.54±3.31	3.71±3.09	0.245	
(<7.0 pg/mL)	6 weeks	8.44±8.53	7.62±4.41	0.809	0.266 (0.038)
	12 weeks	10.50±9.24	4.06±2.42	0.229	0.139 (0.515)
Fibrinogen	baseline	3.72±0.53	3.99±0.46	0.377	
(1.50–4.00 g/L)	6 weeks	4.50±1.17	4.77±0.84	0.365	**0.014** (**0.017**)
	12 weeks	4.65±1.04	4.23±0.49	0.416	**0.037** (0.214)
Factor VIII activity	baseline	169.09±41.51	160.60±42.12	0.756	
(70–150%)	6 weeks	195.45±61.02	218.91±60.77	0.490	0.130 (0.093)
	12 weeks	199.7±67.26	257.09±150.23	0.360	0.169 (**0.017**)
Protein C activity	baseline	107.36±33.99	109.70±12.46	0.145	
(>70%)	6 weeks	108±32.68	106.55±18.67	0.767	0.799 (0.475)
	12 weeks	101.5±27.26	114±19.76	0.084	0.113 (0.540)
Protein S activity	baseline	85.36±12.26	86.80±12.55	0.848	
(>60%)	6 weeks	82.18±15.16	104.36±27.09	**0.036**	0.266 (0.086)
	12 weeks	87.3±14.54	91±10.6	0.549	0.799 (0.507)
von Willebrand factor	baseline	164.09±42.81	174.90±71.14	0.973	
activity (70–130%)	6 weeks	222.27±59.75	201.73±71.76	0.554	**0.013** (0.075)
	12 weeks	209.5±77.35	215.27±75.31	0.883	**0.013** (0.333)
Antithrombin III activity	baseline	92.73±13.72	98.90±11.50	0.191	
(>80%)	6 weeks	96.73±15.31	98.2±9.78	0.944	0.082 (0.779)
	12 weeks	96.4±12.08	96.73±9.51	0.751	0.407 (0.681)

*Between group comparison, Mann-Whitney U test;

**Comparison versus baseline (in brackets p-value of control group), Wilcoxon test.

The minimal mean hepatic threshold dose at 6 weeks after BT (primary endpoint) was significantly higher in the study treatment group than the control (19.1 Gy versus 14.6 Gy, p = 0.011, Table 4) with comparable results with the intention-to-treat analysis (Table 4). Correspondingly, fewer patients in the study treatment group than the control had evidence of fRILI at 6 weeks (45.5% versus 90.9%); this difference was also significant in the intention-to-treat analysis (Table 4). However at 12 weeks after BT (and 4 weeks after cessation of study treatment), these between-group differences were not observed (in neither the per-protocol nor intention-to-treat analyses) for the minimal mean hepatic threshold dose and the proportion of patients with fRILI (Table 4). Results from the optional follow-up at 24 weeks after BT continually showed no between-group differences for the minimal mean hepatic threshold dose and the proportion of patients with fRILI (no change of the proportion of patients with fRILI as compared to 12 weeks follow-up; the minimal mean hepatic threshold dose for treatment group was 20.1 Gy (1 patient missing) and for the control group 21.0 Gy; p>0.05, per-protocol analysis (with comparable results with the intention-to-treat analysis)).

**Table 4:** Minimal mean hepatic tolerance dose (Gy) and evidence of detectable focal radiation-induced liver injury (fRILI) after BT, group comparison.

Variable	Group			p-value(between groups)
**Minimal mean hepatic** **tolerance dose** **(primary endpoint)**		**Dose (Gy)**	**SD**	
At 6 weeks	Control	14.64 [14.15]	4.01 [3.93]	
	Treatment	19.06 [18.46]	3.35 [3.59]	**0.011** [**0.007**]
At 12 weeks	Control	16.38 [16.10]	3.57 [3.60]	
	Treatment	19.04 [18.50]	2.88 [3.11]	0.069 [0.082]
**Detectable fRILI**		**Counts**	**Frequency**	
At 6 weeks	Control	10 [12]	90.9% [92.3%]	
	Treatment	5 [7]	45.5% [53.8%]	**0.022** [**0.027**]
At 12 weeks	Control	10 [12]	90.9% [92.3%]	
	Treatment	10 [12]	90.9% [92.3%]	1.000 [1.000]
				

Per protocol analysis (n = 22); Intention-to-treat analysis (n = 26) in square brackets.

Covariate analyses also showed no influence of recorded covariables on the primary endpoint; only group allocation was significant (Table 5).

**Table 5:** Covariate analysis of minimal mean hepatic tolerance dose 6 weeks after BT (per protocol, n = 22).

Covariate*	p-value(group influence)	p-value(co-variate influence)
Sex (m/f)	**0.015**	0.458
Age (y)	**0.016**	0.864
Weight (kg)	**0.010**	0.117
Height (cm)	**0.011**	0.485
ECOG at baseline (0 and 1 vs 2)	**0.008**	0.310
EQ5D visual analogue score	**0.015**	0.868
History of liver surgery	**0.007**	0.064
Steatosis hepatis	**0.014**	0.845
Diabetes mellitus	**0.015**	0.627
Chemotherapy pre treatment	**0.012**	0.373
Used chemotherapeutic agents		
Oxaliplatin	**0.013**	0.991
Irinotecan	**0.011**	0.327
Biologicals	**0.012**	0.459
Number of treated metastases	**0.013**	0.681
Maximum diamter of metastases (mm)	**0.023**	0.669
Clinical target volume (cm^3^)	**0.013**	0.815
Liver volume (cm^3^)	**0.018**	0.937
Interval from BT to 6 weeks FU (days)	**0.008**	0.258
Liver volume with a dose exposure >10 Gy (%)	**0.013**	0.598
Chemotherapy during follow-up	**0.015**	0.191
Bilirubin baseline	**0.030**	0.401
Albumin baseline	**0.020**	0.784
Aspartate transaminase baseline	**0.025**	0.263
Alanine transaminase baseline	**0.006**	0.092
Cholinesterase baseline	**0.013**	0.425
Gamma glutamyltransferase baseline	**0.012**	0.317
Glutamate dehydrogenase baseline	**0.011**	0.352
International normalized ratio baseline	**0.008**	0.783
Interleukin 6 baseline	**0.030**	0.401
Fibrinogen baseline	**0.002**	0.232
Factor VIII activity baseline	**0.005**	0.615
Protein C activity baseline	**0.004**	0.868
Protein S activity baseline	**0.004**	0.831
von Willebrand factor activity baseline	**0.004**	0.763
Antithrombin III activity baseline	**0.008**	0.261

*Two-way ANOVA for categorical factors, ANCOVA for metric covariables.

EQ5D (as a descriptor of quality of life) and distribution of ECOG performance status were not significantly different at baseline (Table 2) or at any follow-up visit (Table S1). Median overall survival from time of BT on was not different between the groups with 30.0 months (95%CI: 8.7–51.3) in the treatment group and 39.5 months (27.5–51.5) in the control group (p = 0.430).

Safety analyses were conducted in all 30 patients who received BT. The following mild-to-moderate adverse events CTCAEv3 grade 1–2 were reported (in the treatment/control groups) on day 3 after BT: pain (1 patient/1 patient) and fatigue (0/1); at week 6: pain (2/0), fatigue (0/1), nausea (1/0) and diarrhea (2/0); nausea and diarrhea was probably related to PTX or UDCA. One grade 3 subacute bleeding episode from the bile duct, related to BT, occurred in the study treatment group which was successfully managed by endoscopic coagulation.

Analysis of the laboratory data revealed no grade 3/4 changes in bilirubin or albumin. One grade 1 reduction of albumin in the treatment group at 6 weeks was unchanged at week 12. One patient in control group with elevated (grade 1) bilirubin at baseline remained stable throughout follow-up. RILD was not observed on either group.

Laboratory analysis regarding liver-specific and inflammatory/hemostatic parameters found no relevant findings at baseline (Table 3). At week 6, slightly higher gamma-glutamyl-transferase levels and protein-S-activity were recorded in the control group compared with the treatment group. At 6 and 12 weeks, there was slight but significant mean decrease from baseline in cholinesterase in the treatment group. Additionally, mean fibrinogen and von-Willebrand-factor-activity increased significantly from baseline in the treatment group at 6 and 12 weeks; while significant increases from baseline were recorded with mean fibrinogen, factor-VIII-activity and aspartate-transaminase in the control group at 6 weeks.

No correlation between the minimal mean hepatic threshold and liver-specific and inflammatory/hemostatic laboratory values was found at either week 6 or 12 (data not shown).

## Discussion

In this prospective study, we were able to show a significant reduction in fRILI (as measured by hepatobiliary MRI) at 6 weeks after BT of colorectal liver metastases in patients who received low-dose LMWH, PTX and UDCA. Re-assessment of patients at 12 weeks (4 weeks after cessation of study treatment) found that the extent and incidence of fRILI was comparable to the control group, thereby supporting the reliability of our findings. This is further authenticated by the results of the (optional) 24 weeks follow-up. According to our results we believe that we were able to mitigate rather than delay the fRILI by the prophylactic regimen. The finding that the positive effect of the medication to the liver parenchyma as seen at the 6 weeks follow-up vanished after discontinuation of the medication (after 8 weeks) in the 3 months follow-up, make us believe that the fRILI was in fact mitigated in that period. Further on, the extent of the fRILI at 6 weeks in the treatment group and at 3 months (and 6 months) in both groups was less in size compared to the fRILI in the control group at 6 weeks (the peak of the fRILI in our study). Thus, the maximum extent of the fRILI at 6 weeks was skipped in the treatment group as compared to the control group. However, the radiation damage could not be suppressed completely by the prophylactic regimen with a rebound after cessation of the treatment to the level of the control group in later follow-ups. Thus, it is possibly right to assume additionally a delay on the development of the fRILI by the prophylactic regimen. This delay is considered to be advantageous as well since a rapid formation of the fRILI can be delayed (and mitigated) allowing the liver remnant to compensate for the fRILI. However, although appropriately powered, the study should be understood as a pilot due to the small sample size. To compensate for the rebound of the fRILI after cessation of the prophylactic regimen and for a better understanding of the dynamics of the fRILI, a study concept with a prolonged course for the prophylactic regimen is planned.

RILI remains a challenge in the treatment of liver malignancies by radiotherapy (whether percutaneous, interstitial or by radioembolization) because it may eventually translate into RILD or REILD. Further on, life-threatening VOD associated with combined-modality induced liver disease occurs in 5–60% of patients undergoing BMT [18], [23], [26]. For this reason, the potentially protective effects of a number of treatments including low-dose LMWH, PTX and UDCA have been evaluated. Although the efficacy appears equivocal in some studies [14], [15], [16], [17], [18], [19], [20], [21], [28] (Table 1), we determined that the combination of low-dose LMWH, PTX and UDCA appeared to be the most promising option for further evaluation with BT. We believe that our success in showing a benefit in ameliorating fRILI with this combination is based on the following factors: a highly homogeneous patient cohort; attention to patient compliance to the prophylactic regimen; and direct measurement of damage to the liver parenchyma rather than clinical endpoints.

The treatment course of 8 weeks for the medication was determined on the assumption that occurrence of RILD and fRILI peaks around 2 months after radiation-exposure [5], [6], [7], [25]. However, our findings suggest that the radiation-induced injury to the liver structures and cell endothelial continues beyond 8 weeks and that discontinuation of the medication at this time allows the development of a veno-occlusive state/liver cell dysfunction. Endothelial cell damage, which triggers local thrombotic mechanisms, leading to microvascular flow insufficiency, production of cytotoxic substances, and ultimately hepatocellular necrosis, has been thought to be an early event in the development of RILD/VOD [5], [10], [11], [30], [31]. The current evidence indicates that PTX, low-dose LMWH and UDCA may act through a variety of mechanisms to alleviate these effects. PTX, for example, down regulates tumor-necrosis factor-α (TNF-α), a prime suspect in either the initiation or amplification of tissue injury following radiation. PTX also stimulates vascular endothelial production of non-inflammatory prostaglandins of the E- and I-series, enhancing loco-regional blood flow and promoting thrombolysis [16].

LMWHs are assumed to prevent subsequent thrombosis of hepatic venules after endothelial damage and therefore decrease the risk of VOD/RILD [18].

By oral administration of UDCA the concentration of potentially liver toxic hydrophobic bile acids can be reduced [32]. Several *in vitro* studies suggest that potential attenuating effects of UDCA on the pathogenesis of VOD is achieved through the down-regulation of inflammatory cytokine such as TNF-α and interleukin-1 [33]. These cytokines not only induce and amplify liver damage but are also associated with apoptosis in endothelial cells [34] and the development of VOD. UDCA also appears to have a direct effect on programmed-cell death, inhibiting apoptosis and protecting against the membrane damaging effects associated with hydrophobic bile acids in both hepatocytes and non-liver cells [35].

The rationale for this combined treatment approach is based on the assumption that LMWH, PTX and UDCA, which act through a variety of different mechanisms, may act synergistically or in a complimentary fashion to protect the liver [26], [27], [28]; although further study is needed to fully evaluate this hypothesis. However, based on the low toxicity profile of these medications, we believe that this initial approach can be justified. Although the patient numbers are small, the absence of severe toxicities acccords with experience of other published data [15], [16], [17], [19], [20], [21], [28].

Regarding changes of laboratory values, no clinically relevant (grade 3/4) toxicities were observed. The observed slight increases (varying over time and group) of fibrinogen, factor-VIII-activity, protein-S-activity and von-Willebrand-factor-activity correspond most likely to an unspecific increase in acute-phase proteins after radiotherapy or/and to a consequence of radiation-induced endothelial damage of the hepatic veins and sinuses with subsequent platelet aggregation. Regarding the course of liver specific laboratory paramters after BT, it might be argued that the induced fRILI was possibly too small to induce a significant overall increase of these parameters. However, the slight but significant increase of aspartate transaminase in the control group indicates a parenchymal damage. Interestingly, this increase was not seen in the treatment group, indicating a decreased parenchymal damage under preventive medication.

The primary endpoint in our analysis is based on a surrogate i.e. fRILI visualized and quantified using hepatobiliary contrast agent (Gd-EOB-DTPA)-enhanced MRI. Hepatobiliary contrast agents differ from other gadolinium chelates in that they are selectively taken up by functioning hepatocytes through an organic-anion-transporter-polypeptide (mainly OATP1B1 and 3) and excreted into the bile by the multidrug-resistance-protein-2. For Gd-EOB-DTPA, the biliary excretion rate is approximately 50% in humans [36], [37]. Regardless of the mechanism of damage to liver, the hepatobiliary contrast media in functionally altered liver parenchyma is significantly reduced [38]. This is also true for fRILI since a loss of uptake of hepatobiliary contrast media is clearly evident in the liver parenchyma adjacent to the clinical target volume after local radiotherapy (Figure 2) [6], [7]. Importantly, an agreement has been found between the histopathological evidence of fRILI/VOD and loss of hepatocellular uptake of hepatobiliary contrast agent [24].

Unlike the reduced uptake of hepatobiliary contrast agents in sinusoidal-obstruction-syndrome observed after platinum-containing chemotherapy (which is reticular in geometry and generalized all over the liver) [39], the reduced uptake of hepatobiliary contrast media after BT is focal, homogenous and circumferential around the clinical target volume (Figure 1) [6], [7]. Thus, we believe that we can exclude underlying sinusoidal-obstruction-syndrome as a confounder of our results. Additionally, the history of platinum-containing chemotherapy was equal between the groups and without influence on the endpoint.

We suggest that our study results can be transferred to other established radiation treatment methods of liver malignancies such as ^90^Y-radioembolization. According to conversion calculations, the dose ranges in the liver parenchyma associated with ^90^Y-radioembolization and BT are comparable, if re-calculated with respect to the standard fractionation. We therefore hypothesize that preventive treatment approaches against RILD/REILD should be equally effective for both ^90^Y-radioembolization and BT.

## Conclusions

In summary, our results show a highly significant reduction in fRILI after BT of colorectal liver metastases in patients who received low-dose LMWH, PTX and UDCA. Further on, we believe that these findings can be adopted for the prevention of radiation-induced liver damage after other radiotherapeutic approaches as ^90^Y-radioembolization and that further clinical studies in this area are warranted.

## Supporting Information

Table S1 **ECOG, EQ5D dimensions and EQ5D VAS, baseline and follow-up; group comparison (per-protocol only).**
(DOCX)

Checklist S1 **Consort Checklist regarding the present study.**
(DOCX)

Protocol S1 **Study protocol as submitted to the competent authorities.**
(PDF)
