# Supplementary material for: Prospective Randomized Trial of Enoxaparin, Pentoxifylline and Ursodeoxycholic Acid for Prevention of Radiation-Induced Liver Toxicity
Source: PLoS One. 2014 Nov 13;9(11):e112731. doi: 10.1371/journal.pone.0112731 (PMC4231047; doi:10.1371/journal.pone.0112731)
Supplement: Table S1 — ECOG, EQ5D dimensions and EQ5D VAS, baseline and follow-up; group comparison (per-protocol only). (DOCX) [file pone.0112731.s001.docx]

| **Table S1.** |  |  |  |
| --- | --- | --- | --- |
|  |  |  |  |
| ECOG, EQ5D dimensions** and EQ5D VAS****, baseline and follow-up (per-protocol only) | | | |
|  |  |  |  |
| **Variable** | **Medication group (n=11)** | **Control group (n=11)** | **p-value*** |
|  |  |  |  |
| ECOG (grade 0;1;2) baseline (n) | 6;4;1 | 4;5;2 | 0.370 |
| ECOG (grade 0;1;2) 6 weeks (n) | 7;4;0 | 7;4;0 | 1.000 |
| ECOG (grade 0;1;2) 3 months (n) | 4;5;2 | 5;6;0 | 0.401 |
| EQ5D Mobility (grade 1;2;3;4;5) baseline (n) | 10;1;0;0;0 | 11;0;0;0;0 | 0.317 |
| EQ5D Mobility (grade 1;2;3;4;5) 6 weeks (n) | 9;2;0;0;0 | 10;1;0;0;0 | 0.544 |
| EQ5D Mobility (grade 1;2;3;4;5) 3 months (n) | 6;5;0;0;0 | 9;2;0;0;0 | 0.180 |
| EQ5D Self-care (grade 1;2;3;4;5) baseline (n) | 11;0;0;0;0 | 11;0;0;0;0 | 1.000 |
| EQ5D Self-care (grade 1;2;3;4;5) 6 weeks (n) | 11;0;0;0;0 | 11;0;0;0;0 | 1.000 |
| EQ5D Self-care (grade 1;2;3;4;5) 3 months (n) | 10;1;0;0;0 | 10;1;0;0;0 | 1.000 |
| EQ5D Usual activities (grade 1;2;3;4;5) baseline (n) | 11;0;0;0;0 | 10;1;0;0;0 | 0.317 |
| EQ5D Usual activities (grade 1;2;3;4;5) 6 weeks (n) | 10;1;0;0;0 | 10;1;0;0;0 | 1.000 |
| EQ5D Usual activities (grade 1;2;3;4;5) 3 months (n) | 10;1;0;0;0 | 9;2;0;0;0 | 0.544 |
| EQ5D Pain/discomfort (grade 1;2;3;4;5) baseline (n) | 7;4;0;0;0 | 10;1;0;0;0 | 0.140 |
| EQ5D Pain/discomfort (grade 1;2;3;4;5) 6 weeks (n) | 6;5;0;0;0 | 9;2;0;0;0 | 0.180 |
| EQ5D Pain/discomfort (grade 1;2;3;4;5) 3 months (n) | 4;7;0;0;0 | 8;3;0;0;0 | 0.094 |
| EQ5D Anxiety/depression (grade 1;2;3;4;5) baseline (n) | 10;1;0;0;0 | 11;0;0;0;0 | 0.147 |
| EQ5D Anxiety/depression (grade 1;2;3;4;5) 6 weeks (n) | 9;2;0;0;0 | 7;4;0;0;0 | 0.350 |
| EQ5D Anxiety/depression (grade 1;2;3;4;5) 3 months (n) | 8;3;0;0;0 | 9;2;0;0;0 | 0.619 |
| EQ5D total baseline (median sum per individual)*** | 6 | 5 | 0.270 |
| EQ5D total 6 weeks (median sum per individual)*** | 6 | 6 | 0.519 |
| EQ5D total 3 months (median sum per individual)*** | 6 | 6 | 0.729 |
| EQ5D VAS baseline (%, mean, standard deviation) | 72.4 (14.6) | 76.4 (13) | 0.438 |
| EQ5D VAS 6 weeks (%, mean, standard deviation) | 77.3 (12.3) | 76.8 (15.1) | 0.898 |
| EQ5D VAS 3 months (%, mean, standard deviation) | 75.8 (12.7) | 85.9 (13.8) | 0.076 |
| *comparing the groups, continuous data compared by Mann-Whitney U test, frequency data compared by Pearson’s chi square test | | | |
| **regarding mobility, self-care, usual activities, pain/discomfort and anxiety/depression; each dimension scored from 1 (=no problems) to 5 (=extreme problems) | | | |
|  |  |  |  |
| ***sum of scores from each EQ5D dimension with 5 indicating no problems and 25 extreme problems | | | |
| ****visual analogue score regarding self rated health, range from 0 (worst) to 100 (best) | | | |
