# Supplementary material for: Prospective Randomized Trial of Enoxaparin, Pentoxifylline and Ursodeoxycholic Acid for Prevention of Radiation-Induced Liver Toxicity
Source: PLoS One. 2014 Nov 13;9(11):e112731. doi: 10.1371/journal.pone.0112731 (PMC4231047; doi:10.1371/journal.pone.0112731)
Supplement: Protocol S1 — Study protocol as submitted to the competent authorities. (PDF) [file pone.0112731.s003.pdf]

Evaluation of the preventive effect of enoxaparin, pentoxifylline and ursodeoxycholic acid to radiation induced liver toxicity after brachytherapy of liver metastases from colorectal carcinoma, assessed in a prospective randomised trial.

"Eldorado"

**Clinical study phase:**	II	**Version / Date:**	2.3 / 01.10.08
**EudraCT No.:**	2008-002985-70	**Status:**	
**Author:**	Max Seidensticker, Robert Damm, Ricarda Rühl, Prof. Dr. med. Jens Ricke		

The study will be conducted in compliance with the protocol, ICH-GCP and any applicable regulatory requirement.

The approval of this study protocol is documented in a separate signature document

Confidential

The information provided in this document is strictly confidential and is intended solely for the guidance of the clinical investigation. Reproduction or disclosure of this document – whether in part or full – to parties not associated with the clinical investigation or its use for any other purpose without prior written consent of the sponsor is not permitted.

Synopsis

Study title	Evaluation of the preventive effect of enoxaparin, pentoxifylline and ursodeoxycholic acid to radiation induced liver toxicity after brachytherapy of liver metastases from colorectal carcinoma, assessed in a prospective randomised trial	
Short title	“Eldorado”	
Clinical study phase	II	
Study objectives	To evaluate whether a combination regimen of pentoxifylline, ursodeoxycholic acid and low dose low molecular weight heparin (enoxaparin) provides a protective effect on the liver parenchyma after HDR brachytherapy.	
Test product	enoxaparin, pentoxifylline, ursodeoxycholic acid	
Reference product	None	
Indication	Patients with colorectal liver metastases scheduled for brachytherapy for clinical reasons	
Diagnosis and main criteria for inclusion	Liver metastases from colorectal carcinoma	
Study design	Randomised, prospective, parallel group, open label	
Methodology	All patients receive a single fraction CT/MRI-guided HDR-brachytherapy of colorectal liver metastases using Iridium-192. The follow-up consists of 4 MRI controls of the abdomen using the hepatocyte-specific contrast agent Primovist (Gd-EOB-DTPA) after 3 days, 6 weeks, 3 months and 6 months as well as blood samples and a questionnaire taken the same time.<br>Within the study, 22 patients are given low dose low molecular weight heparin, pentoxifylline and ursodeoxycholic acid for 8 weeks starting with the preinterventional day. Another 22 patient will receive the standard therapy without the medication.<br>After completion of the follow-up, MRI volume data of the lesion will be acquired and compared to the dosimetric treatment plan. Blood samples are tested for liver-specific and inflammatory laboratory parameters.	
Type of control	Standard therapy	
Planned study dates	FPFV	
	LPLV	
Planned number of study centers	1	
Planned number of countries	1	
Number of patients	44 valid patients	
Primary variable	HDR-brachytherapy isodose that marks the border between damaged and functioning liver tissue (as defined by Primovist-enhanced MR imaging)	
Plan for statistical analysis	According to the statistical part of the protocol.	

Table of Contents

Introduction **8**

1.1 Background 8

1.2 Rationale for the study 8

1.3 Benefit-risk assessment 9

2 Study Objectives 10

2.1 Primary objective 10

2.2 Secondary objective 10

3 Overview of methodology and design 10

3.1 Study design 10

3.2 Study organization 10

3.3 Type of control 10

3.4 Justification of the design 10

3.5 Protocol adherence 10

4 Study population 11

4.1 Eligibility / description 11

4.1.1 Inclusion criteria 11

4.1.2 Exclusion criteria 11

4.2 Recruitment 11

4.3 Withdrawal and replacement criteria for treatment 12

4.4 Withdrawal and replacement criteria for assessment 12

4.5 Patient identification 12

5 Study drug 12

5.1 Study drug and comparators 12

5.2 Identity of study drug(s) 13

5.3 Rationale for unusual or novel approaches 13

5.4 Dosage and administration 13

5.5 Treatment assignment 14

5.6 Blinding 14

5.7 Packaging and labelling 14

5.8 Drug logistics and accountability 14

5.8.1 Supply, storage, dispensation and return 14

5.8.2 Drug accountability 14

5.9 Treatment compliance 14

6 Therapies other than study drug 14

6.1 Prior and concomitant medication 15

6.2 Post-study therapy 15

7 Schedule of evaluations and visit description 15

7.1 Schedule of evaluations 15

7.2 Visit description 16

7.3 Flowchart 17

7.4 Follow-up period 17

7.5 End of study 17

8 Procedures and variables 17

8.1 Description of the primary analysis set and if applicable the cases to be excluded from the primary analysis17

8.2 Subgroup analysis, if planned 18

8.3 Primary target variables 18

8.4 Secondary target variables 18

8.5 Safety 18

8.5.1 Baseline findings 18
8.5.2 Adverse events 19
8.5.3 Further safety 25

8.6 Appropriateness of procedures / measurements 25

9 Statistical methods and determination of sample size 25

9.1 List of variables and population characteristics 25

9.2 Interim analyses 26

9.3 Determination of sample size 26

9.4 Randomization/Stratification 26

10 Data handling and quality assurance 26

10.1 Data recording 26

10.2 Monitoring 27

10.3 Data processing 27

10.4 Auditing 28

10.5 Archiving 28

11 Premature termination of study 28

12 Ethical and legal aspects 28

12.1 Ethical and legal conduct of the study 28

12.2 Patient information and consent 28

12.3 Financing 29

12.4 Publication policy 29

12.5 Compensation for health damage of patients / insurance 29

13 References 30

14 Further reading 31

List of abbreviations

3D	Three dimensional
ADR	adverse drug reaction
AE	adverse event
ALAT	Alanine aminotransferase
AMG	Arzneimittelgesetz
ASAT/ALAT	Aspartate aminotransferase
AT3	Antithrombin 3
ChE	Cholinesterase
CRA	clinical research associate
CRF	case report form
CRO	contract research organization
CT	Computed tomography
CTCAE	Common Terminology Criteria for Adverse Events
ECG	Electrocardiogram
ECOG	Eastern Cooperative Oncology Group
FAS	full analysis set
FPFV	first patient, first visit
gamma-GT	Gamma glutamyl transferase
GCP	Good Clinical Practice
Gd EOB DTPA	Gadolinium ethoxybenzyl diethylenetriaminepentaacetic acid
GLDH	Glutamatdehydrogenase
Gy	Gray
H	hour
HCC	hepatocellular carcinoma
HDR	High dose rate
ICD	International Statistical Classification of Diseases and Related Health Problems
ICH	International Conference on Harmonization
IEC	independent ethics committee
INR	International normalized ratio
IRB	institutional review board
ITF	investigator trial file
IV	intravenous
Kg	kilograms
LKP	“Leiter der klinischen Prüfung”
LPLV	last patient, last visit
Min	minute(s)
ML	milliliter
MRI	Magnetic resonance imaging
n.a.	not applicable
NCI	National Cancer Institute
p.i.	post injection
PAI	Plasminogen activator inhibitor
PP	per protocol
RECIST	Response Evaluation Criteria In Solid Tumors
RILD	Radiation induced liver disease
SAE	serious adverse event
SDV	source data verification
SmPC	Summary of Product Characteristics
SUSAR	suspected unexpected serious adverse reaction
TGF ß1	Transforming growth factor beta 1
TMF	trial master file
TNM	TNM Classification of Malignant Tumors
VOD	Veno-occlusive disease

VWF	Von Willebrand factor

Study administrative structure

Sponsor	**Otto-von-Guericke-University**<br>Faculty for Medicine<br>represented by the dean<br><br>**Prof. Dr. med. Hermann Josef ROTHKÖTTER**<br>Otto-von-Guericke-University<br>Faculty for Medicine<br>Leipziger Str.44<br>39120 Magdeburg, Germany<br><br>Phone: +49-(0)391-67-15750<br>Fax: +49-(0)391-67-15749<br>e-mail: hermann-josef.rothkoetter@med.ovgu.de
Principal Investigator	**Prof. Dr. Jens RICKE**<br>Director, Clinic for Radiology and Nuclear Medicine<br>Universitätsklinikum Magdeburg<br>Leipziger Str. 44<br>39120 Magdeburg, Germany<br><br>Phone: +49 391 67 13030<br>Fax: +49 391 67 13029<br>e-mail: jens.ricke@med.ovgu.de
Co-Investigator	**Max SEIDENSTICKER**<br>Physician<br>Clinic for Radiology and Nuclear Medicine<br>Universitätsklinikum Magdeburg<br>Leipziger Str. 44<br>39120 Magdeburg, Germany<br><br>Phone: +49 391 67 13030<br>Fax: +49 391 67 13029<br>e-mail: max.seidensticker@med.ovgu.de
Co-Investigator	**Ricarda RÜHL**<br>Physician<br>Clinic for Radiology and Nuclear Medicine<br>Universitätsklinikum Magdeburg<br>Leipziger Str. 44<br>39120 Magdeburg, Germany<br><br>Phone: +49 391 67 13030<br>Fax: +49 391 67 13029<br>e-mail: ricarda.rühl@med.ovgu.de

Introduction

1.1 Background

For patients with liver malignancies which are untreatable by surgery or chemotherapy, loco-regional application of irradiation like HDR-brachytherapy provides a new promising option. HDR-brachytherapy is performed by inserting catheters through the liver parenchyma precisely into a certain liver malignancy by using CT or MR imaging. After the correct placement of the catheters, three-dimensional irradiation planning follows to calculate the application dose and to spare contiguous organs. The constant position of the catheter ensures no effect of breathing movements during irradiation, here with Iridium-192. Patients receive a single fraction therapy irradiating the liver lesions with a high dose. Even though precise radiation planning permits the delivery of a sufficient target dose to the tumor lesions, damage of surrounding healthy tissue cannot be completely avoided. Nearby liver parenchyma typically shows a dysfunction in follow-up MRI studies which is characterized by a diminished circumscribed uptake of the hepatocyte-specific MRI contrast agent Primovist (Gd-EOB-DTPA) as well as edema in the former target zone. By merging dosimetry data with follow-up-MRI it is possible to identify the threshold dose for irreversible liver damage.
Histopathological studies suggest a veno-occlusive disease as the underlying pathology of irreversible liver damage after irradiation. It is characterised by damage of the sinusoidal and central-venous endothelium with fibrin deposition, congestion, and occlusion leading to hepatocyte dysfunction. Later an irreversible fibrosis is observed. [1,2]
This study intends to compare the occurence of radiation injury in patients after local irradiation therapy of hepatic metastasis of colorectal carcinoma prophylactically treated with pentoxifylline, ursodeoxycholic acid and low dose low molecular weight heparin (enoxaparin) compared to a group of patients without protective medication (current clinical standard). The diminished uptake of Primovist (Gd-EOB-DTPA) following HDR-brachytherapy is supposed to indicate impaired hepatocyte function. [3-5]
The cumulative application of three drugs intends to create a maximum, therapy relevant effect. [6]

1.2 Rationale for the study

A preventive effect of pentoxifylline, ursodeoxycholic acid and low dose low molecular weight heparin on pathological processes in healthy tissue after irradiation is described in clinical studies on percutaneous liver irradiation and on bone marrow transplantation. However, data remains inconclusive. [7-14]

This exploratory study aims at assessing whether a protective effect of the combination of pentoxifylline, ursodeoxycholic acid, and low dose low molecular weight heparin can be demonstrated in a limited number of patients with liver metastases of colorectal cancer after HDR brachytherapy.

1.3 Benefit-risk assessment

This study includes Magnetic Resonance Imaging (MRI) and the application of the MR contrast agent Primovist as a part of the standard follow-up. There are very few risks associated with MRI scans. The changing radiofrequencies and magnetic fields theoretically can produce heat, but this is not known to be associated with relevant side effects. The risk of the injection of MR contrast agents is considered to be low.

Risks additional to those of the standard therapy include possible side effects of the medication for the patients of the treatment arm as well as possible interactions between the study drugs and any other drugs taken by those patients.
Interactions between the three study drugs are not conceivable. Interactions with any other drug can not be obviated. For security reasons, the family physician receives information about the study and the applied drugs to consider possible interactions with the patients other drugs.

According to the SmPC of Pentoxifylline, the most common adverse drugs reactions include nausea, vomiting and diarrhoea. Other reactions like headaches, sleeping disorders and flushing are reported as well. In very rare cases, intrahepatic cholestasis, thrombocytopenia, bleedings and hypersensitivity were observed. Pentoxifylline should be used with caution in patients with severe coronary artery disease and impaired renal function (both conditions are excluded in this study).
The SmPC of ursodeoxycholic acid include reports of interaction with cholestyramine, charcoal, cholestipol and certain antacides as well as oestrogen-rich drugs. As adverse drug reaction, diarrhoea may occur rarely.
Enoxaparin is contraindicated in patients with acute bacterial endocarditis, active major bleedings and high risk of uncontrolled haemorrhage as well as active gastric or duodenal ulcerations. Application of any other anticoagulant or thrombolytics must be discontinued during heparine therapy. According to the SmPC, caution should be exercised in patients with renal impairment, low body weight, in elderly patients, patients scheduled for a spinal puncture and when a history of heparin-induced thrombocytopenia is known. Enoxaparin is reported to cause bleedings and rarely hyperkalaemia as well as vasculitis. Major haemorrhage including retroperitoneal and intracranial bleeding are known. If bleedings occure, the origin must be investigated and treated appropriately. Enoxaparin may cause an asymptomatic and reversible increase of platelet counts and liver enzymes. Additionally, the subcutaneous injection may lead to pain, mild local irritation and haematoma. Exceptional cases of skin necrosis have been reported.

This study aims at assessing the assumed protective action of a combination regimen after single fraction HDR brachytherapy. If successful, the results of this study have the potential to reduce the probability of severe side effects in these critically ill patients. Participating patients may have a personal benefit as they may experience less hepatocytic damage in the treatment arm. In light of the relatively low rate of side effects, the exclusion of patient groups with possible vulnerability for side-effects and the promise of improving the benefit-risk ratio of an accepted brachytherapy regimen, the benefit-risk ratio for this study is regarded as favorable. Patients in whom a theoretical risk of study medication and/or Primovist-enhanced MRI cannot be a priori excluded are not allowed to enter this study.

2 Study Objectives

2.1 Primary objective

To assess if a combination regimen of pentoxifylline, low dose low molecular weight heparin and ursodeoxycholic acid provides a preventive effect regarding irradiation damage to liver parenchyma after HDR-brachytherapy.

2.2 Secondary objective

- To evaluate the relation between hepatocyte dysfunction as assessed in Primovist-enhanced MRI and changes in liver-specific and inflammatory laboratory values.
- To evaluate the quality of live comparing both patient groups using the EQ-5D questionnaire and ECOG performance status.
- To assess the safety of the combination regimen of pentoxifylline, low dose low molecular weight heparin, and ursodeoxycholic acid given after HDR brachytherapy.

3 Overview of methodology and design

3.1 Study design

Randomised, prospective, parallel group, open label.

3.2 Study organization

Mono-center study.

3.3 Type of control

Group of 22 patients receiving no additional medication (current clinical standard).

3.4 Justification of the design

For this exploratory study the single center approach with a limited number of patients is regarded as appropriate. The required number of patients can be recruited in the university of Magdeburg, Germany, within a reasonable time period. The in- and exclusion criteria and all study procedures are selected to assure that a homogeneous patient population is included.

3.5 Protocol adherence

Strict adherence to all specifications laid down in this protocol is required for all aspects of the study conduct; the investigator may not modify or alter the procedures described in this protocol. If protocol modifications are necessary, all alterations that are not solely of an administrative nature require a formal protocol amendment (see section 12.1 for the involvement of IEC(s)/IRB(s)).

If an investigator has deviated from the protocol in order to eliminate an immediate hazard to patients or for other inevitable medical reasons, the investigator shall document all such deviations, including the reasons thereof, and submit the document to the sponsor and the head of the medical institution as applicable.

4 Study population

4.1 Eligibility / description

4.1.1 Inclusion criteria

- Age 18 to 80
- If female, postmenopausal or surgically sterilized
- Liver metastases from colorectal carcinoma scheduled for a CT/MRI-guided single-fraction interstitial HDR brachytherapy
- Non-cirrhotic liver
- Life expectancy longer than 6 months
- Willing and able to undergo all study procedures
- Having voluntarily provided written and fully informed consent

4.1.2 Exclusion criteria

- Women who are pregnant, lactating or who are of childbearing potential
- Liver cirrhosis
- Hepatitis B
- Hepatitis C
- Patients being clinically unstable
- Uncooperative, in the investigator’s opinion
- Having been previously enrolled in this study
- Participating in another therapy-modulating clinical trial
- Contraindication for MRI
- Contraindication or hypersensitivity to one or more components of Primovist, Enoxaparin, Ursodeoxycholic acid and/or Pentoxifylline
- Any prior irradiation therapy of the liver
- Close affiliation with the investigational site; e.g. a close relative of the investigator
- Severe coronary artery disease
- Autoimmune diseases
- Acute bacterial endocarditis
- Active major bleedings and high rish of uncontrolled haemorrhage
- Patients with severe or moderate renal impairment (GFR below 60 mL/min/1.73 m^2 according to the MDRD or Cockroft-Gault formula, calculated from a creatinine value obtained within 1 week before each planned Primovist-enhanced MR examination)

4.2 Recruitment

Potential study patients meeting the in- and exclusion criteria are asked in a personal dialogue during the admission one day prior to brachytherapy. The patient information leaflet is taken as the basis for the discussion.

Afterwards, patients are granted 24 hours time for consideration regarding their participation in the study. Signature of the informed consent must be done prior to the single fraction brachytherapy the next day.

4.3 Withdrawal and replacement criteria for treatment

Every patient has the right to refuse further participation in the study at any time and without providing any reasons (see also section 12.2). A patient's participation is to be terminated immediately upon his/her request. The investigator should seek to obtain the reason and record this on the CRF.

Patients may be withdrawn from the study at any time at the discretion of the investigator; the reason should be fully documented on the CRF. Should the patient, during the course of the study, develop conditions which would have prevented his/her entry into the study according to the exclusion criteria, he/she must be withdrawn immediately. The reasons are to be fully documented on the CRF. The termination of an individual's participation should be considered in case of a SAE or considerable worsening of the patient's clinical symptoms.

At the discretion of the Sponsor or the Principal Investigator, the entire study or individual parts of the study may be canceled for medical or administrative reasons. In case of premature termination or suspension of the study, the Principal Investigator will promptly inform the regulatory authorities and IEC/IRBs of the termination or suspension and the corresponding reason.

4.4 Withdrawal and replacement criteria for assessment

Patients not completing the follow-up period up to at least 3 months, patients with interruption of medication and patients with an progression of treated intrahepatic metastases within 3 months according to RECIST criteria will be excluded from the primary efficacy analysis. Available data for these patients will be reported only. These patients will be replaced in order to obtain a number of 44 valid patients for the analysis.

4.5 Patient identification

Patients of each study group (group A for the medication group and group B for the comparison group) will be numbered separately. Patients of group A will be assigned a 2-digit patient number in ascending order starting with 01 preceded by A. Patients of group B will be assigned a 2-digit patient number in ascending order starting with 01 preceded by B.

Examples:
Patient A03 – 3rd patient of group A
Patient B09 – 9th patient of group B

5 Study drug

5.1 Study drug and comparators

Pentoxifylline:
Trental® 400mg as approved for marketing
manufacturer: Sanofi-Aventis

Ursodeoxycholic acid:
Ursofalk® 250mg as approved for marketing
manufacturer: Falk Pharma

Enoxaparin (low molecular weight heparin):
Clexane® 40mg as approved for marketing

manufacturer: Sanofi-Aventis

5.2 Identity of study drug(s)

A complete record of batch numbers and expiry dates of all study medication will be maintained in the TMF.

Pentoxifylline

- modified release tablet
- strength 400mg
- dose 3x400mg/day
- oral application
- administration for 8 weeks since the evening of the day of intervention
- CAS 6493-05-6
- ATC C04AD03

Ursodeoxycholic acid

- white, opaque, hard gelatine capsule
- strength 250mg
- dose 3x250mg/day
- oral application
- administration for 8 weeks since the evening of the day of intervention
- CAS 128-13-2
- ATC A05AA02

Enoxaparin (Low moleculare weight heparin)

- solution for injection
- strength 40mg
- dose 1x40mg/day
- subcutaneous injection
- administration for 8 weeks since the evening of the day of intervention
- CAS 9005-49-6
- ATC B01AB05

5.3 Rationale for unusual or novel approaches

Not applicable.

5.4 Dosage and administration

Ursodeoxycholic acid is administered for 8 weeks since the evening of the day of intervention. Dosage is 250mg given three times daily (morning, noon, evening).
Pentoxifylline is given for 8 weeks since the evening of the day of intervention with a dose of 400mg applied three times daily (morning, noon, evening).
Enoxaparin with a dose of 40mg is injected subcutaneously once a day for 8 weeks since the evening of the day of intervention after the HDR-brachytherapy.

5.5 Treatment assignment

Participating patients are assigned either to the medication group or to the non-medication group by randomisation. The randomization list is provided to the investigator before start of the study.

5.6 Blinding

Volumetry of the lesion of hepatocyte dysfunction as indicated in contrast enhanced MRI using Gd-EOB-DTPA (Primovist) is performed blinded

5.7 Packaging and labelling

Packaging of the drugs is original. Open label use.

5.8 Drug logistics and accountability

5.8.1 Supply, storage, dispensation and return

Patients of the medication group receive the drugs during the inpatient stay from the responsible physician. At discharge from hospital the drugs for the remaining period are handed out with a thorough instruction.
All drugs are supplied by the Clinic for Radiology and Nuclear Medicine of the University of Magdeburg.

5.8.2 Drug accountability

n.a.

5.9 Treatment compliance

The compliance of all patients of the medication group is evaluated during the personal dialogue at the second and third visit.

In addition, the blood level of anti-Xa activity is evaluated four hours after injection. A blood sample is taken during the second visit (6 weeks after brachytherapy) to observe the compliance of enoxaparin administration.
The compliance regarding pentoxifylline and ursodeoxycholic acid intake will be appraised on the basis of compliance with enoxaparin (anti-Xa activity, see above).

Insufficient compliance of drug application leads to patient´s withdrawal from the analysis and further study-specific medication is stopped. Follow-up is then performed according to the standard therapy program.
Insufficient compliance is marked by an Anti-Xa activity lower than 0,1 IU/ml (international units per milliliter, measured up to 4h after last injection) or when a patient reports an interruption of the whole drug administration for more than one day a week twice.

6 Therapies other than study drug

Patients undergo the standard therapy of HDR brachytherapy at the Clinic for Radiology and Nuclear Medicine at the University Hospital of Magdeburg.

6.1 Prior and concomitant medication

The study does not consider any prior or concomitant medication besides the drugs applicated to the medication subgroup. Patients may receive concomitant therapy during the study as required. Any concomitant medication at baseline and during the study as well as any changes made in concomitant medication will be recorded on the CRF.

6.2 Post-study therapy

Post-study therapy will follow routine clinical care.

7 Schedule of evaluations and visit description

7.1 Schedule of evaluations

22 patients undergoing CT- or MR-guided HDR brachytherapy receive the combination regimen as stated in section **5.4**.
Another group of 22 patients is treated according to the standard procedure of HDR brachytherapy without the periinterventional medication (current clinical standard).

The study consists of 6 visits.

1. visit
One day prior to brachytherapy, including admission and education about the study during a personal dialogue on the basis of the patient information.
Preinterventional MRI and laboratory evaluation (standard therapy).

2. visit
The day of brachytherapy.
Signing of the informed consent before the intervention. Laboratory evaluation of blood samples for the preinterventional laboratory values prior to the intervention.
Medication starts for the treatment group after the intervention including a personal instruction of heparine injection.

3. visit
End of inpatient stay 3 days after brachytherapy including a personal dialogue.
MRI and laboratory evaluation including study specific laboratory parameters.
Evaluation of response according to RECIST.

4. visit
Follow-up after 6 weeks including MRI, blood samples and questioning.
Compliance is checked by Anti-Xa activity (see **5.9**) and a personal dialogue.
Evaluation of response according to RECIST.

5. visit
Follow-up after 3 months including MRI, blood samples and questioning.
Compliance checked by a personal dialogue.
Medication ended 2 weeks ago.
Evaluation of response according to RECIST.

6. visit
Follow-up after 6 months including MRI, blood samples and questioning.
Evaluation of response according to RECIST.

7.2 Visit description

Patients have an inpatient stay one day prior to 3 days after brachytherapy.
During the inpatient stay, visits are done one day prior, the day of and 3 days after brachytherapy. Follow-up visits are planned at 6 weeks, 3 months and 6 months after brachytherapy.

A visit contains a personal dialogue between the patient and an investigator including a check for adverse events and evaluation of the actual quality of life using the EQ-5D questionnaire and the ECOG performance status.
During the fourth and fifth visit, patients taking the study drugs are asked for the compliance.

Simultaneously, blood samples are taken at each visit for chemical analysis of parameters according to the standard follow up procedure:

- bilirubin
- ASAT/ALAT
- albumin
- ChE
- gamma-GT
- GLDH
- INR

Furthermore, values for bilirubin, ASAT/ALAT, gamma-GT and INR are graded for toxicity according to the National Cancer Institute (NCI) CTCAE3.0 (Common Terminology Criteria for Adverse Events).

Additionally, the following study specific parameters are being analysed:

- fibrinogen
- fibrin monomer
- factor VIII
- IL 2 + 6
- PAI
- protein c + s
- vWF
- AT3

For the study specific parameters, two additional blood containers with 4,5ml citrate blood and 8,5ml serum are taken as well as another container for security reasons in each case.
Thus, a total of 26ml blood are taken additionally for study purposes.
Additional probes are deepfrozen, collected and will be analysed after the finished recruitment. All additional probes will be destroyed two years after finishing the clinical trial.

MRI is done as a part of the standard follow-up using the following sequences:

before contrast agent application
T1 axial native & fat saturated
T2 axial native & fat saturated

after contrast agent application (Gd-EOB-DTPA/Primovist® i.v. (0,1 mg / kg body weight)):
dynamic T1 axial THRIVE
T2 axial fat saturated)

10 – 15 min post contrast agent application :
T1 sagittal
T1 axial fat saturated

20 min after contrast agent application:
T1 axial THRIVE

For the study specific MRI volumetry, dynamic axial T1 THRIVE (Exclusion of tumor progression / local recurrence) and T1 axial THRIVE 20 min after application of Gd-EOB-DTPA (Area of hepatocyte dysfunction) are mandatory.

7.3 Flowchart

<table>
<tr><td></td><td>preparation</td><td>intervention</td><td>follow-up</td><td></td><td></td><td></td><td></td></tr>
<tr><td>Weeks</td><td>0</td><td></td><td>1</td><td>6</td><td>8</td><td>12</td><td>24</td></tr>
<tr><td>Visit</td><td>1</td><td>2</td><td>3</td><td>4</td><td></td><td>5</td><td>6</td></tr>
<tr><td>education</td><td>X</td><td></td><td></td><td></td><td></td><td></td><td></td></tr>
<tr><td>informed consent</td><td></td><td>X</td><td></td><td></td><td></td><td></td><td></td></tr>
<tr><td>patient history</td><td>X</td><td></td><td></td><td></td><td></td><td></td><td></td></tr>
<tr><td>physical examination</td><td>X</td><td></td><td></td><td></td><td></td><td></td><td>X</td></tr>
<tr><td>HDR brachytherapy</td><td></td><td>X</td><td></td><td></td><td></td><td></td><td></td></tr>
<tr><td>medication for med. subgroup</td><td></td><td colspan="4">|--|</td><td></td><td></td></tr>
<tr><td>variables</td><td></td><td></td><td></td><td></td><td></td><td></td><td></td></tr>
<tr><td>MRI liver</td><td>X</td><td></td><td>X</td><td>X</td><td></td><td>X</td><td>X</td></tr>
<tr><td>lab tests</td><td>X</td><td></td><td>X</td><td>X</td><td></td><td>X</td><td>X</td></tr>
<tr><td>EQ-5D</td><td>X</td><td></td><td>X</td><td>X</td><td></td><td>X</td><td>X</td></tr>
<tr><td>safety</td><td></td><td></td><td></td><td></td><td></td><td></td><td></td></tr>
<tr><td>adverse events</td><td></td><td>X</td><td>X</td><td>X</td><td></td><td>X</td><td>X</td></tr>
</table>

7.4 Follow-up period

The follow-up period contains the stated MRI examinations and laboratory tests of the blood samples, all according to the standard therapy. Additionally, ECOG performance status and EQ-5D questionnaires are completed at each visit and the additional blood samples are taken. The response of the intrahepatic metastases according to RECIST is evaluated as well.

7.5 End of study

LPLV.

8 Procedures and variables

8.1 Description of the primary analysis set and if applicable the cases to be excluded from the primary analysis

MRI volumetry of diminished uptake of contrast agent Gd-EOB-DTPA (Primovist) shown in the axial T1 THRIVE sequence 20 minutes after injection is done blinded by two independent radiologists. The volumes will be quantified corresponding to the isodoses of the irradiation plans. The midpoint, median and standard deviation of the resulting isodoses from each patients MRI at a certain point of time are calculated and compared between the two patient groups.
Graphical presentation is done by a histogram showing the midpoint and standard deviation. All patients withdrawn from the study are excluded from the primary analysis.

8.2 Subgroup analysis, if planned

No subgroup analysis is planned.

8.3 Primary target variables

HDR-brachytherapy isodose that marks the border between irreversibly damaged and functioning liver tissue (as defined by Primovist-enhanced MR imaging).

Volume data will be acquired by volumetry using the computer program OsiriX for MacOS X. By identifying the irreversibly damaged volume in every layer of the axial T1 THRIVE image, 3D data can be calculated and correlated to a specific isodose when merged with the 3D irradiation treatment plan.

Example

A lesion 3 months after HDR-brachytherapy extends up to the 10,2 Gy isodose. The threshold dose is, therefore, 10,2 Gy; i.e., the liver parenchyma exposed to at least 10,2 Gy of absorbed dose shows a diminished uptake of Primovist and is regarded as irreversibly damaged.

8.4 Secondary target variables

- Change in laboratory values (treatment group vs. control group), NCI CTCAE score
- Adverse drug reactions
- Quality of live (EQ-5D questionnaire, ECOG performance status)
- Side effects of HDR brachytherapy

8.5 Safety

8.5.1 Baseline findings

8.5.1.1 Definition of baseline findings

Definition of baseline finding

A baseline finding is defined as any untoward medical condition in a study patient who has signed the informed consent form but not yet received the first dose of the study drug. This includes conditions stabilized by treatment. By definition, a baseline finding cannot be causally related to study drug; however, it may be causally related to the study (e.g., caused by study-conduct-related investigations).

Differentiation between medical/surgical history and baseline findings

Conditions which started *before signature of informed consent* and for which no symptoms or treatment are present until the first administration of study drug (e.g., seasonal allergy without acute complaints) are recorded as medical/surgical history.

Conditions which started *before signature of informed consent* and for which symptoms or treatment are present between signature of informed consent and first administration of study drug (e.g., allergic pollinosis) are recorded as baseline findings.

Differentiation between baseline findings and adverse events
Conditions (e.g., abnormal physical examination findings, symptoms, diseases, laboratory, ECG) present *before the first administration of study drug* will be documented as baseline findings.

Conditions which started or deteriorated *after the first administration of study drug* will be documented as adverse events.

8.5.1.2 Categories, assessments and documentation of adverse events

8.5.1.3 Serious baseline findings

Definition

Baseline findings will be regarded as serious if they meet the criteria used for defining SAEs (see Section 8.5.2.5).

Serious baseline findings will be reported on the SAE form described in section 8.5.2.5.

8.5.2 Adverse events

8.5.2.1 Definition of adverse event

The definition below follows ICH-GCP (see also ICH Guideline for clinical Safety Data Management: Definitions and Standards for Expedited Reporting):

Adverse event (AE)
Any untoward medical occurrence in a patient or clinical investigation subject administered a pharmaceutical product and which does not necessarily have a causal relationship with this treatment. An AE can therefore be any unfavourable and unintended sign (including an abnormal laboratory finding), symptom, or disease temporally associated with the use of a medicinal (investigational) product, whether or not considered related to the medical (investigational) product.

By definition, for this study, all AEs are regarded as “treatment emergent”; i.e., not seen before treatment or, if already present before treatment, worsened after start of treatment.

8.5.2.2 Categories for adverse event assessment

All AEs will be assessed and documented by the investigator according to the categories detailed below.

Seriousness
For each AE, the seriousness must be determined according to the criteria given in Section 8.5.2.5.

Intensity

The intensity of an AE is classified according to the following categories, taking into account the possible range of the intensity of the event:

- Mild
- Moderate
- Severe
- Optional category

Main pattern

The main pattern of the AE is to be documented as follows:

Every drug administration: Events that occur in a clear time relationship to every study drug administration

Intermittent: Regular or irregular repeating events that are clearly of the same kind and same cause, but not clearly time related to study drug administration

Continuous: Events that are continuously present within the whole time period which is covered by the form, but not clearly time related to study drug administration

Other: All other patterns, need to be specified in the following text field

Study drug action

Any potential study drug action to resolve the AEs is to be documented as follows

- Drug withdrawn
- Dose reduced
- Dose not changed
- Other action (entered in free text, e.g., 'dose interrupted', 'dose interrupted and re-started')

Drug treatment

Non-drug treatment

Causal relationship to study drug

The possible causal relationship between the AE and the administration of the study drug is classified according to the following definitions:

None:	The time course between administration of the study drug and occurrence or worsening of the AE rules out a causal relationship.<br><br>*and / or*<br><br>Another cause is confirmed and no indication of involvement of the study drug in the occurrence / worsening of the AE exists.
Unlikely:	The time course between administration of the study drug and occurrence or worsening of the AE makes a causal relationship unlikely.<br><br>*and / or*<br><br>The known effects of the study drug or of the substance class provide no indication of

	involvement in the occurrence / worsening of the AE and another cause adequately explaining the AE is known.<br><br>*and / or*<br><br>Regarding the occurrence / worsening of the AE a plausible causal chain may be deduced from the known effects of the study drug or the substance class, but another cause is much more probable.<br><br>*and / or*<br><br>Another cause is confirmed and involvement of the study drug in the occurrence / worsening of the AE is unlikely.
Possible:	Regarding the occurrence / worsening of the AE, a plausible causal chain may be deduced from the pharmacological properties of the study drug or the substance class, but another cause just as likely to be involved is also known.<br><br>*or*<br><br>Although the pharmacological properties of the study drug or the substance class provide no indication of involvement in the occurrence / worsening of the AE, no other cause gives adequate explanation.
Probable:	The pharmacological properties of the study drug or of the substance class,<br><br>*and / or*<br><br>The course of the AE after dechallenge and, if applicable, after rechallenge,<br><br>*and / or*<br><br>Specific tests (e.g., positive allergy test, antibodies against study drug / metabolites) suggest involvement of the study drug in the occurrence / worsening of the AE, although another cause cannot be ruled out.
Definite:	The pharmacological properties of the study drug or of the substance class,<br><br>*and*<br><br>The course of the AE after dechallenge and, if applicable, after rechallenge,<br><br>*or*<br><br>Specific tests (e.g., positive allergy test, antibodies against study drug / metabolites) indicate involvement of the study drug in the occurrence / worsening of the AE and no indication of other causes exists.

‘Related’ AEs comprise the categories ‘possible’, ‘probable’ and ‘definite’.

Causal relationship to study conduct

The possible causal relationship between the AE and any study-conduct-related procedures and activities required by the protocol is classified according to the following definitions:

None:	The nature of the AE or the time course between study-conduct-related procedures and activities and occurrence or worsening of the AE rules out a causal relationship<br><br>*and / or*<br><br>Another cause is confirmed and no indication of involvement of the study conduct in the occurrence / worsening of the AE exists.

Unlikely:	The time course between study-conduct-related procedures and activities and occurrence or worsening of the AE makes a causal relationship unlikely.<br><br>*and / or*<br><br>The known risks of the study-conduct-related procedures and activities provide no indication of involvement in occurrence / worsening of the AE and another cause adequately explaining the AE is known.<br><br>*and / or*<br><br>Regarding the occurrence / worsening of the AE, a plausible causal relationship may be deduced from the known risks of the study-conduct-related procedures and activities, but another cause is much more probable.<br><br>*and / or*<br><br>Another cause is confirmed and involvement of the study-conduct-related procedures and activities in the occurrence / worsening of the AE is unlikely.
Possible:	Regarding the occurrence / worsening of the AE, a plausible causal relationship may be deduced from the known risks of the study-conduct-related procedures and activities, but another cause just as likely to be involved is also known.<br><br>*or*<br><br>Although the known risks of the study-conduct-related procedures and activities provide no indication of involvement in the occurrence / worsening of the AE, no other cause gives adequate explanation.
Probable:	Regarding the occurrence / worsening of the AE, a plausible causal relationship is suggested by the known risks of the study-conduct-related procedures and activities<br><br>*or*<br><br>No other cause is just as likely.
Definite:	Regarding the occurrence / worsening of the AE, a plausible causal relationship is suggested by the known risks of the study-conduct-related procedures and activities and other causes can be ruled out.

‘Related’ AEs comprise the categories ‘possible’, ‘probable’ and ‘definite’.

Outcome

The outcome of the AE is to be documented as follows:

- Recovered / resolved
- Recovering / resolving
- Not recovered / not resolved
- Recovered / resolved with residual effects
- Fatal
- Unknown.

8.5.2.3 Assessments and documentation of adverse events

AEs are assessed by indirect questioning.

8.5.2.4 Expected adverse events

Expected disease-related AEs

Adverse events that can be caused by liver metastases of a colorectal carcinoma include:

- Elevation of liver enzymes
- Cholestasis
- Pain (right upper quadrant)
- Compression of vena cava inferior
- Weight-loss
- Anemia
- Dizziness
- Fatigue
- Liver enlargement

Expected conduct-related AEs

Adverse events that can be caused by HDR-brachytherapy include:

- Fever
- Liver abscess
- Cholangitis
- Elevation of liver enzymes
- Bleedings
- Pain
- Pleural effusion
- Jaundice
- Ascites

Expected ADRs

The definition below follows ICH-GCP (see also ICH Guideline for Clinical Safety Data Management: Definitions and Standards for Expedited Reporting):

Adverse Drug Reaction (ADR)
A response to a drug which is noxious and unintended and which occurs at doses normally used in man for prophylaxis, diagnosis, or therapy of diseases or for modification of physiological function.

Expected side effects

Pentoxifylline
frequently (1-10%):
nausea, emesis, diarrhoea
occasionally (0,1-1%):
arrhythmia, erythema, urticaria, tremor, fever, headache, insomnia, conjunctivitis
infrequently (<0,1%):
cholestasis, hypotension, angina pectoris, elevation of liver enzymes, bleedings, thrombocytopenia, aplastic anaemia, convulsions, epidermal necrolysis, aseptic meningitis

Patients having autoimmune diseases are considered predisposed.

Ursodeoxycholic acid

frequently (1-10%):
pulpily faeces

Enoxaparin
erythema, exanthema, angioedema, hyperthermia, vasculitis, hyperkalaemia, mild asymptomatic thrombozytopenia during the first days of application, asymptomatic reversible elevation of platelet counts and liver enzymes,
pain, mild local irritation and haematoma due to subcutaneous injection
infrequently:
thrombocytosis, leucopenia, bradycardia, headache, systemic allergic reactions, skin necrosis at the injection side

Unexpected ADRs
Adverse drug reactions are to be considered unexpected if they add significant information on the specificity or intensity of an expected ADR. The expectedness of an AE/ADR shall be determined by the sponsor according to the SmPC.

The term “unexpected”, as used in this definition, refers to an ADR currently not included in the SmPC; it does not imply that this ADR was not anticipated because of the pharmacological properties of the study drug.

8.5.2.5 Serious adverse events

Definition of Serious Adverse Event
The following SAE definition is based on ICH guidelines and the final rule issued by the Food and Drug Administration (FDA) and effective 06 Apr 1998. It is to be applied to both, AEs (defined in Section 8.4.2.1) and baseline findings (defined in Section 8.4.1.1).

An SAE is classified as any untoward medical occurrence that at any dose

- Results in death, or
- Is life threatening, or
- Requires inpatient hospitalization or prolongation of existing hospitalization, or
- Results in persistent or significant disability / incapacity, or
- Is a congenital anomaly / birth defect

The term ‘life threatening’ in the definition refers to an event in which the patient was at risk of death at the time of the event, it does not refer to an event which hypothetically might have caused death if it were more severe.

Medical and scientific judgment should be exercised in deciding whether it is appropriate to report an AE as serious also in other situations, such as important medical events that may not be immediately life threatening or result in death or hospitalization but may jeopardize the patient or may require intervention to prevent one of the other outcomes listed in the definition above. These should also usually be considered serious. Examples of such events are intensive treatment in an emergency room or at home for allergic bronchospasm; blood dyscrasias or convulsions that do not result in inpatient hospitalization; or development of drug dependency or drug abuse.

Actions and reporting obligations in case of serious adverse events

In case of serious adverse events, notification of IEC/IRB, authorities, co-investigators and any other person (involved in this study) for whom the knowledge of the SAE is important, is in the responsebility of the principal investigator

8.5.3 Further safety

8.5.3.1 Laboratory evaluations

Blood samples are taken at each visit according to the standard therapy.
Laboratory parameters being analysed according to Section 7.1.

Responsible laboratory:

Institute for Clinical Chemistry and Pathological Biochemistry
Universitätsklinikum Magdeburg
Leipziger Str. 44
39120 Magdeburg, Germany

Phone: +49 391 67 13900
Fax: +49 391 67 13902
e-mail: ikcp@med.ovgu.de

8.5.3.2 Physical examination

Physical examination is performed during the first and the last visit of each patient.

8.5.3.3 12-lead ECG

No ECG is planned.

8.5.3.4 Vital signs

n.a.

8.6 Appropriateness of procedures / measurements

The volume of the radiation induced liver lesions is defined by the uptake of the hepatocyte specific MRI contrast agent Gd-EOB-DTPA (Primovist). Previous studies have shown the characteristics of radiation-induced liver lesions as defined by hepatocyte specific contrast agents.[15]
For laboratory evaluations, liver enzymes (e.g. ASAT/ALAT) and function/synthesis parameters (e.g. bilirubin, albumin) were chosen to reflect the liver function. To appraise inflammation and fibrosis induced by irradiation, blood parameters (e.g. fibrinogen) are included.
Quality of life is measured by EQ-5D questionnaire and ECOG performance status to rate effects of HDR-brachytherapy and the study medication.

9 Statistical methods and determination of sample size

9.1 List of variables and population characteristics

- Age
- Gender
- Weight
- Height
- Previous and concomitant medication

- Surgical history
- History of malignant disease including treatment
- MRI volume data
- Laboraty parameters
- Quality of life
- Treatment compliance for medication group

9.2 Interim analyses

An interim analysis is scheduled when 11 patients per group finished the 3 months follow-up. The interim analysis will contain all statistical methods that will be used for the final analysis. If the interim anlysis show a futility to proceed, i.e. inverse results than hypothesized, a termination of the study is mandatory.

9.3 Determination of sample size

A previous study characterizing the radiation-induced liver lesion by MRI volumetry found a minimal threshold dose of 9,9Gy (standard deviation 2,3Gy) 6 weeks after intervention in a similar population of patients without the study medication.[15]
We expect an increase of the threshold dose to at least 12Gy under medication.
A sequential test with 2 stages according to the Pocock design is used.
A difference of 2,1Gy with a standard deviation of 2,3Gy yields a total of 22 observations per group with an interim analysis after 11 observations per group when α=0,025 and power 1-β=0,8.

Responsible statistician:

PD Dr. rer. nat. Siegfried Kropf
Institute for Biometry and Medical Informatics
Universitätsklinikum Magdeburg
Leipziger Str. 44
39120 Magdeburg, Germany

Phone: +49 391 67 13524
Fax: +49 391 67 13536
e-mail: siegfried.kropf@med.ovgu.de

9.4 Randomization/Stratification

Patients are randomized for one of the subgroups, either receiving the drugs or not.
Sealed envelopes are handed out randomised by the study support office.

10 Data handling and quality assurance

10.1 Data recording

Data recording is done with an electronic database. A double data entry is applied to verify data collection from the printed CRF.

The following variables are captured (if applicable for each visit):

- Patient group
- Patient number
- Initials
- Birth date
- Gender
- Date of information about the study
- Date of informed consent
- Visit dates
- ICD code for colorectal cancer
- Date of diagnosis
- TNM classification
- Neoadjuvant chemotherapy or radiotherapy
- Surgical history
- Adjuvant chemotherapy or radiotherapy
- Details on liver metastases
- Palliative chemotherapy
- Concomitant diseases
- Concomitant medication
- Details on HDR brachytherapy
- Clinical status
- MRI sequences
- MRI volume data
- Response according to RECIST
- Laboratory parameters
- NCI CTCAE grading of specific laboratory parameters
- Result of EQ-5D questionnaire
- ECOG performance status
- Study drug administration
- Treatment compliance
- Side effects
- Adverse events
- Serious adverse events
- Information on withdrawal

10.2 Monitoring

Monitoring is done by the study support office of the Clinic for Radiology and Nuclear Medicine, University of Magdeburg.
Each patient’s CRF is checked for completeness after each follow-up. SDV is done by a doubla data entry within the electronic database.
Access to the source data must be provided to the sponsor and to health authorities upon request.

10.3 Data processing

Assessment of NCI CTCAE grades for laboratory parameters is done by an automatic algorithm within the electronic database.

10.4 Auditing

A member of the sponsor's (or a designated CRO's) quality assurance unit may arrange to visit the investigator in order to audit the performance of the study at the study site and the study documents originating there. The auditor(s) will usually be accompanied by the CRA. The investigator will be informed about the outcome of the audit.

In addition, inspections by health authority representatives – including foreign authorities – and IEC(s)/IRB(s) are possible at any time. The investigator is to notify the sponsor of any such inspection immediately.

10.5 Archiving

The sponsor and the investigator/medical institution shall, in every case, retain essential documents relating to this trial for at least 15 years after its completion. They shall retain the documents for a longer period if required by other applicable regulatory requirements or by a separate agreement between the sponsor and the investigator. Essential documents shall be archived in such a way that ensures that they are readily available upon authorities' request.

Patient (hospital) files will be archived according to local regulations and in accordance with the maximum period of time permitted by the hospital, institution or private practice. The ITF (investigator's trial file) is not to be destroyed without the sponsor's approval. The investigator's contract will contain all regulations relevant for the study center.

11 Premature termination of study

At the discretion of the sponsor or the Principal Investigator, the entire study may be discontinued for medical or administrative reasons. In case of premature termination the investigators, IRB/IECs and regulatory authorities will be informed by the Principal Investigator.

12 Ethical and legal aspects

12.1 Ethical and legal conduct of the study

The planning and conduct of this clinical study are subject to national laws. The study will be conducted in accordance with the protocol, the ethical principles that have their origin in the Declaration of Helsinki and ICH-GCP.

The study protocol and any amendments are to be reviewed by an IEC/IRB and, if applicable, health authorities before implementation.

12.2 Patient information and consent

All relevant information on the study will be summarized in an integrated patient information and consent sheet provided by the sponsor or the study center. A sample patient information and informed consent form is provided as a document separate to this protocol.

Based on this patient information sheet, the investigator will explain all relevant aspects of the study to each patient, before his/her entry into the study (i.e., before examinations and procedures associated with selection for the study are performed).

The investigator will also mention that written approval of the IRB/IEC has been obtained.

Each patient will have ample time and opportunity to ask questions and will be informed about the right to withdraw from the study at any time without any disadvantage and without having to provide reasons for this decision.
Following this informative discussion, the patient will be asked if he/she is willing to sign and personally date a statement of informed consent, which includes consenting to the processing of his/her data as explained in the patient information sheet. Only if the patient voluntarily agrees to sign the informed consent form and has done so, may he/she enter the study. Additionally, the investigator will personally sign and date the form, too. The patient will receive a duplicate of the signed and dated form.

The signed informed consent statement is to remain in the ITF or, if locally required, in the patient’s note/file of the medical institution.

The investigator will document on the CRF the time and date of obtaining informed consent.

In the event that informed consent is obtained on the date that baseline study procedures are performed, the study record or patient's clinical record must clearly show that informed consent was obtained prior to these procedures.

The informed consent form and any other written information provided to patients will be revised whenever important new information becomes available that may be relevant to the subject’s consent, or there is an amendment to the protocol which necessitates a change to the content of the patient information and/or the written informed consent form. The investigator will inform the patient of changes in a timely manner and will ask the subject to confirm his/her participation in the study by signing the revised informed consent form. Any revised written informed consent form and written information must receive the IRB/IEC's approval/favourable opinion in advance of use.

A sample of the integrated patient information and consent sheet is provided as a separate document.

12.3 Financing

Each investigator (including principal and/or any subinvestigators; as well as their spouses and dependent children) who is directly involved in the treatment or evaluation of research subjects has to provide a financial disclosure according to all applicable legal requirements. All relevant documentation will be filed in the TMF and/or ITF, as appropriate.

12.4 Publication policy

The results of this study will be published.

12.5 Compensation for health damage of patients / insurance

Where required by the laws and regulations of the country in which the study is performed, insurance of patients against health impairment occurring as a result of participation in the study will be set up in accordance with said laws and regulations. All relevant documentation regarding such insurance will be filed in the TMF and/or ITF, as appropriate.

13 References

(1) Fajardo LF, Colby TV. Pathogenesis of veno-occlusive liver disease after radiation. Arch Pathol Lab Med. 1980;104:584-588.

(2) Fajardo LF. The pathology of ionizing radiation as defined by morphologic patterns. Acta Oncol. 2005;44:13-22.

(3) Pascolo L, Cupelli F, Anelli PL et al. Molecular mechanisms for the hepatic uptake of magnetic resonance imaging contrast agents. Biochem Biophys Res Commun. 1999;257:746-752.

(4) Reimer P, Schneider G, Schima W. Hepatobiliary contrast agents for contrast-enhanced MRI of the liver: properties, clinical development and applications. Eur Radiol. 2004;14:559-578.

(5) Reimer P, Rummeny EJ, Shamsi K et al. Phase II clinical evaluation of Gd-EOB-DTPA: dose, safety aspects, and pulse sequence. Radiology. 1996;199:177-183.

(6) Shulman HM, Hinterberger W. Hepatic veno-occlusive disease--liver toxicity syndrome after bone marrow transplantation. Bone Marrow Transplant. 1992;10:197-214.

(7) Attal M, Huguet F, Rubie H et al. Prevention of hepatic veno-occlusive disease after bone marrow transplantation by continuous infusion of low-dose heparin: a prospective, randomized trial. Blood. 1992;79:2834-2840.

(8) Bianco JA, Appelbaum FR, Nemunaitis J et al. Phase I-II trial of pentoxifylline for the prevention of transplant-related toxicities following bone marrow transplantation. Blood. 1991;78:1205-1211.

(9) Essell JH, Thompson JM, Harman GS et al. Pilot trial of prophylactic ursodiol to decrease the incidence of veno-occlusive disease of the liver in allogeneic bone marrow transplant patients. Bone Marrow Transplant. 1992;10:367-372.

(10) Forrest DL, Thompson K, Dorcas VG, Couban SH, Pierce R. Low molecular weight heparin for the prevention of hepatic veno-occlusive disease (VOD) after hematopoietic stem cell transplantation: a prospective phase II study. Bone Marrow Transplant. 2003;31:1143-1149.

(11) Ohashi K, Tanabe J, Watanabe R et al. The Japanese multicenter open randomized trial of ursodeoxycholic acid prophylaxis for hepatic veno-occlusive disease after stem cell transplantation. Am J Hematol. 2000;64:32-38.

(12) Or R, Nagler A, Shpilberg O et al. Low molecular weight heparin for the prevention of veno-occlusive disease of the liver in bone marrow transplantation patients. Transplantation. 1996;61:1067-1071.

(13) Rosenthal J, Sender L, Secola R et al. Phase II trial of heparin prophylaxis for veno-occlusive disease of the liver in children undergoing bone marrow transplantation. Bone Marrow Transplant. 1996;18:185-191.

(14) Ruutu T, Eriksson B, Remes K et al. Ursodeoxycholic acid for the prevention of hepatic complications in allogeneic stem cell transplantation. Blood. 2002;100:1977-1983.

(15) Ricke J, Seidensticker M, Ludemann L et al. In vivo assessment of the tolerance dose of small liver volumes after single-fraction HDR irradiation. Int J Radiat Oncol Biol Phys. 2005;62:776-784.

14 Further reading

Ricke J, Wust P, Wieners G et al. Liver malignancies: CT-guided interstitial brachytherapy in patients with unfavorable lesions for thermal ablation. J Vasc Interv Radiol. 2004;15:1279-1286.

Ricke J, Wust P, Stohlmann A et al. [CT-Guided brachytherapy. A novel percutaneous technique for interstitial ablation of liver metastases]. Strahlenther Onkol. 2004;180:274-280.

Sutherland LM, Williams JA, Padbury RT et al. Radiofrequency ablation of liver tumors: a systematic review. Arch Surg. 2006;141:181-190.

Lawrence TS, Robertson JM, Anscher MS et al. Hepatic toxicity resulting from cancer treatment. Int J Radiat Oncol Biol Phys. 1995;31:1237-1248.

Reed GB, Jr., Cox AJ, Jr. The human liver after radiation injury. A form of veno-occlusive disease. Am J Pathol. 1966;48:597-611.

Farthing MJ, Clark ML, Sloane JP, Powles RL, McElwain TJ. Liver disease after bone marrow transplantation. Gut. 1982;23:465-474.

Jacobs P, Miller JL, Uys CJ, Dietrich BE. Fatal veno-occlusive disease of the liver after chemotherapy, whole-body irradiation and bone marrow transplantation for refractory acute leukaemia. S Afr Med J. 1979;55:5-10.

Kim TH, Panahon AM, Friedman M, Webster JH. Acute transient radiation hepatitis following whole abdominal irradiation. Clin Radiol. 1976;27:449-454.

Lewin K, Millis RR. Human radiation hepatitis. A morphologic study with emphasis on the late changes. Arch Pathol. 1973;96:21-26.

Shulman HM, McDonald GB, Matthews D et al. An analysis of hepatic venocclusive disease and centrilobular hepatic degeneration following bone marrow transplantation. Gastroenterology. 1980;79:1178-1191.

Shulman HM, Gown AM, Nugent DJ. Hepatic veno-occlusive disease after bone marrow transplantation. Immunohistochemical identification of the material within occluded central venules. Am J Pathol. 1987;127:549-558.

Bearman SI, Hinds MS, Wolford JL et al. A pilot study of continuous infusion heparin for the prevention of hepatic veno-occlusive disease after bone marrow transplantation. Bone Marrow Transplant. 1990;5:407-411.

Carreras E, Bertz H, Arcese W et al. Incidence and outcome of hepatic veno-occlusive disease after blood or marrow transplantation: a prospective cohort study of the European Group for Blood and Marrow Transplantation. European Group for Blood and Marrow Transplantation Chronic Leukemia Working Party. Blood. 1998;92:3599-3604.

Park SH, Lee MH, Lee H et al. A randomized trial of heparin plus ursodiol vs. heparin alone to prevent hepatic veno-occlusive disease after hematopoietic stem cell transplantation. Bone Marrow Transplant. 2002;29:137-143.

Essell JH, Schroeder MT, Harman GS et al. Ursodiol prophylaxis against hepatic complications of allogeneic bone marrow transplantation. A randomized, double-blind, placebo-controlled trial. Ann Intern Med. 1998;128:975-981.

Attal M, Huguet F, Rubie H et al. Prevention of regimen-related toxicities after bone marrow transplantation by pentoxifylline: a prospective, randomized trial. Blood. 1993;82:732-736.

Clift RA, Bianco JA, Appelbaum FR et al. A randomized controlled trial of pentoxifylline for the prevention of regimen-related toxicities in patients undergoing allogeneic marrow transplantation. Blood. 1993;82:2025-2030.
